# Longitudinal Impacts of Forest Loss on *Bartonella* and Hemotropic *Mycoplasma* Dynamics in Vampire Bats Within a Fragmented Habitat

**DOI:** 10.1111/mec.70466

**Published:** 2026-07-11

**Authors:** Lauren R. Lock, Kristin E. Dyer, Dmitriy V. Volokhov, Anni Yang, M. Brock Fenton, Nancy B. Simmons, Daniel J. Becker

**Affiliations:** ^1^ School of Biological Sciences University of Oklahoma Norman Oklahoma USA; ^2^ Center for Biologics Evaluation & Research U.S. Food & Drug Administration Silver Spring Maryland USA; ^3^ Department of Geography and Environmental Sustainability University of Oklahoma Norman Oklahoma USA; ^4^ Department of Biology Western University London Canada; ^5^ Division of Vertebrate Zoology, Department of Mammalogy American Museum of Natural History New York New York USA

**Keywords:** bartonellae, *Desmodus rotundus*, hemoplasmas, land‐use change

## Abstract

Habitat fragmentation can negatively impact wildlife, including increasing infectious disease risk. We assessed spatiotemporal changes in pathogen dynamics in vampire bats ( *Desmodus rotundus* ) in response to habitat fragmentation using general linear mixed models to investigate the influence of site, year, and tree cover on *Bartonella* spp. and hemotropic *Mycoplasma* spp. (hemoplasmas) infection risk in bats in one large and one small forest fragment in Belize across seven years. While *Bartonella* infections were more likely in the final years of the study regardless of site, hemoplasma infection likelihood was not significantly different across years or sites. *Bartonella* infections were associated with increased forest loss in the large fragment only, whereas hemoplasma infections were not associated with forest loss. The effects of site, year, and forest loss on infection likelihood varied by pathogen genotype despite low model explanatory power. Neither site nor year was associated with bartonellae, but one genotype was positively associated with tree cover. Two hemoplasmas were influenced by year with differing trends: one genotype was negatively associated with tree loss across sites, while another was positively associated with forest loss at the small fragment only. Both pathogens were similarly influenced by bat demographics and showed instances of infection status and genotype switching. Our work suggests that the effects of habitat fragmentation on infection risk depend on both the pathogen and specific genotype, complicating expectations of how environmental change affects wildlife disease dynamics. Efforts to mitigate infectious disease impacts in fragmented systems should be tailored to specific pathogens of concern.

## 1 Introduction

Habitat fragmentation is a process in which an undisturbed habitat is broken into multiple patches. Because it is almost always coupled with habitat loss, it is difficult to differentiate between effects that are due to loss and effects due to fragmentation (Laurance 2008; Didham et al. 2012). While no two ecosystems or species are exactly alike in their response to fragmentation, impacts on biodiversity are most often detrimental (Haddad et al. 2015). Island Biogeography Theory (IBT) is commonly used to predict the effects of habitat fragmentation on biodiversity (Laurance 2008). According to IBT, if a habitat patch is considered to be analogous to an island, diversity is expected to be lower in small, isolated patches compared to large, easily accessible patches (MacArthur and Wilson 1967). The IBT can also be extended to predict the effects of habitat fragmentation on infectious disease dynamics (Reperant 2010; Shaw et al. 2024). This application deviates from the typical expectations of IBT—pathogens should be able to capitalise on the effects of fragmentation on hosts, allowing pathogens to more easily invade small, isolated habitat patches. However, outcomes may differ between pathogens, as pathogen responses to habitat fragmentation are largely dependent upon host responses (i.e., alterations to host richness, abundance, and density; host immunity; host matrix use; Faust et al. 2018; Becker, Albery, et al. 2020) and transmission strategy (Froeschke et al. 2013).

Tropical forests globally have been subjected to exceptionally high rates of habitat fragmentation compared to subtropical, temperate, and boreal regions from 2000 to 2020 (Ma et al. 2023). For example, Ma and colleagues found that tropical forests contained ~12%–29% more area with decreased forest cover and increased fragmentation during this period than subtropical, temperate, or boreal forests (Ma et al. 2023). In the Neotropics, this deforestation imperils an area containing at least one‐third of global biodiversity (Raven et al. 2020) and eight of the world's 34 biodiversity hotspots (Mittermeier et al. 2011). Additionally, habitat fragmentation increases the risk of infectious disease emergence from wildlife by reducing resource availability. Less access to food, shelter, and mates in turn alters wildlife behaviour and can promote physiological stress, increasing the likelihood of pathogen shedding (Plowright et al. 2021; Becker, Dyer, Lock, Fenton, et al. 2025).

Bats (order Chiroptera) are popular and epidemiologically relevant models for studying infectious disease dynamics in wildlife, as they are known reservoirs of a number of zoonotic pathogens, including but not limited to rabies, Nipah, and Hendra viruses (Banyard et al. 2011; Halpin et al. 2011; Letko et al. 2020). Additionally, as the only mammal capable of flight, bats have the potential to disperse pathogens across moderate to long distances (Voigt et al. 2017). Common vampire bats (*Desmodus rotundus*, hereafter ‘vampire bats’) occur throughout the Neotropics and are often abundant in areas that have been disturbed for agricultural purposes, making this species of particular interest to studies of zoonotic pathogens and habitat fragmentation. As vampire bats are dietary specialists that subsist entirely on blood (primarily on that of mammals), the species thrives in close proximity to livestock, especially cattle and pigs (Turner 1975; Delpietro et al. 1992; Bobrowiec et al. 2015). This leads to an increased potential for contact with domestic animals and humans compared to other bat species. Vampire bats are highly social and aggregate in colonies that range in size from a dozen to hundreds of individuals (Trajano 1996; Streicker et al. 2012), creating ample opportunity for density‐dependent pathogen transmission between bats (Lloyd‐Smith et al. 2005). Their social interactions include contact behaviours that can facilitate density‐dependent transmission, such as aggressive encounters between males or frequency‐dependent transmission, such as allogrooming, a high degree of parental care in females and ‘friendships’ between bats that often involve the sharing of regurgitated blood meals (Wilkinson 1984; Yarlagadda et al. 2021; Carter 2024). Vampire bat home ranges vary from ~2 to 5 km (Burns and Crespo 1975; Trajano 1996) but movements of up to 54 km have also been recorded (Delpietro et al. 1992), indicating the potential for pathogen dispersal across moderate distances.

While much of the research pertaining to infectious diseases in vampire bats focuses on rabies virus due to the potential for transmission to humans or domestic animals via blood feeding (Heckley et al. 2023), vampire bats also carry blood‐borne bacterial pathogens such as *Bartonella* spp. (Becker, Bergner, et al. 2018) and hemotropic mycoplasmas (hemoplasmas) (Volokhov et al. 2017). Bartonellae and hemoplasmas in vampire bats have most often been studied in the context of zoonoses, as some lineages can infect humans. For example, ‘*Candidatus* Bartonella mayotimonensis’ has been detected in several bat species (Veikkolainen et al. 2014; Lilley et al. 2017) and implicated in human cases of endocarditis (Lin et al. 2010), while ‘*Candidatus* Mycoplasma haematohominis’, reported to cause hemolytic anaemia in humans (Steer et al. 2011), shares high genetic similarity to the 16S rRNA gene from hemoplasmas detected in Neotropical bats sympatric with vampire bats (Volokhov et al. 2023). However, many *Bartonella* and hemoplasma genotypes are considered to be host‐specific (Pitcher and Nicholas 2005; Frank et al. 2018), with vampire bats hosting at least 29 lineages of bartonellae (Bai et al. 2011; Becker, Bergner, et al. 2018; Becker, Czirják, et al. 2018; Andre et al. 2019) and three lineages of hemoplasmas (Becker, Speer, et al. 2020). Although both infections are likely facilitated by the species' highly social nature and by parasitism by bat flies and fleas (Judson et al. 2015; Volokhov et al. 2017), current evidence suggests *Bartonella* is spread primarily through arthropod vectors (Judson et al. 2015; McKee et al. 2026), while bat‐to‐bat contact is likely the predominant driver of hemoplasma transmission (Willi et al. 2007). While their prevalence within vampire bat populations makes *Bartonella* and hemoplasmas ideal for studying the effects of habitat fragmentation on infectious disease dynamics in wildlife (Volokhov et al. 2017; Becker, Bergner, et al. 2018), differences in transmission and multiple lineages likely mean that responses to fragmentation may not be uniform across or within pathogens.

Because habitat fragmentation is an ongoing process and its effects are often not immediately observable, longitudinal studies are needed to capture patterns in infectious disease dynamics over time (Heckley et al. 2023). To assess temporal changes in pathogen dynamics in bats impacted by habitat fragmentation, we compared the prevalence of all bartonellae and hemoplasmas, as well as that of each pathogen genotype, between vampire bats in two habitat patches within a fragmented forest system in northern Belize across seven years. We also quantified the amount of tree cover lost within the area between and surrounding both habitats (hereafter ‘the matrix’) during the final five years of this study. We then tested the following hypotheses: (1) infection risk will be higher in the smaller habitat patch, because pathogens should more readily invade small, isolated habitats where hosts are more negatively impacted by fragmentation; (2) increased infection rates will be detected in the later years of the study, as the consequences of habitat fragmentation can take years to become observable; and (3) higher infection risk will be associated with less tree cover in the matrix due to host stressors associated with habitat fragmentation (i.e., decreased resource availability). Additionally, we expected that the direction or magnitude of effect could vary by lineage within each pathogen due to variation in transmission strategies or within‐host dynamics.

## 2 Methods

### 2.1 Study Areas

**FIGURE 1:**
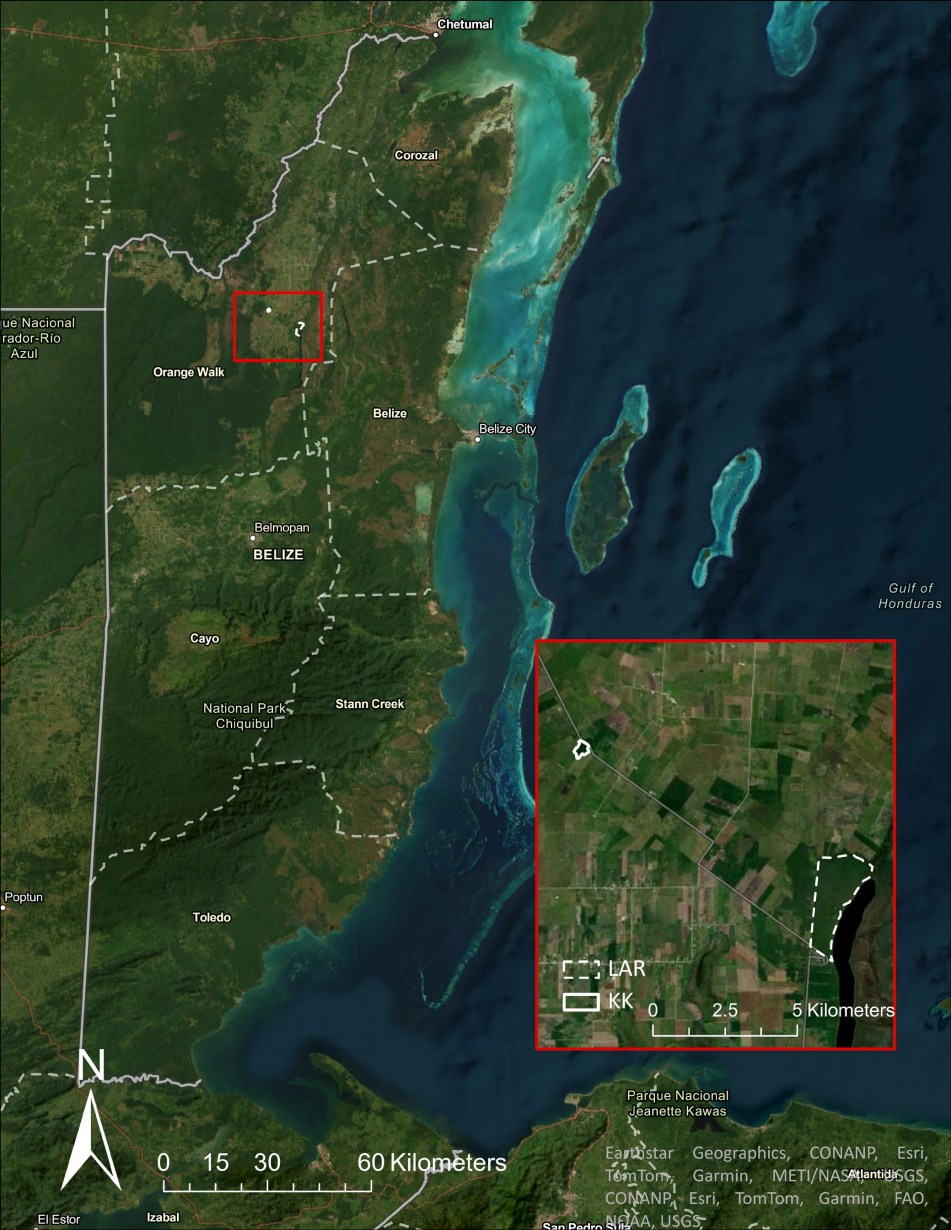
Location and land cover of the Lamanai Archaeological Reserve (LAR) and Ka'Kabish Archaeological Site (KK) bat sampling sites in the Orange Walk District of Belize.

To test all three hypotheses, we sampled vampire bats in the Orange Walk District of Belize, specifically within a preserved forest, the Lamanai Archeological Reserve (LAR; 17.76512, −88.65200) and a preserved forest fragment, Ka'Kabish Archeological Site (KK; 17.81455, −88.73075) (Figure 1). The LAR is a ~450 ha semi‐deciduous forest composed mostly of secondary growth within a protected reserve. Ka'Kabish is a ~45 ha remnant forest fragment bordered by agricultural fields. These sites were connected historically by contiguous forest, but they have since been separated by roughly 10 km of forest fragments and cleared land over about the last twenty years (Becker et al. 2021; Herrera et al. 2018; Ingala et al. 2019).

### 2.2 Bat Sampling

Vampire bats were captured using mist nets over a period of 6–13 nights during each year (six nights per year from 2015 to 2017, 13 nights per year during 2018, 2019, 2021, and 2022). Samples were collected in April and May of each year except for 2020 and 2021. Sampling did not occur in 2020 due to the COVID‐19 pandemic but was resumed in November 2021. Samples were not collected from KK in 2021 due to the abandonment of a major roost by the resident bats. Vampire bats were captured at this site in the subsequent year but at lower numbers. After capture, all bats were held in individual clean cloth bags until processing. We recorded the sex, reproductive status, age class, weight, forearm length, and the unique identification number of each bat prior to sampling. We took blood samples by lancing the propatagial vein using 23‐gauge sterile needles and collecting blood in heparinised capillary tubes, which were then transferred to Whatman FTA cards (Qiagen). Samples were stored and shipped at ambient temperature until long‐term storage at −20°C. We marked each bat with an incoloy wing band with a unique alphanumeric code (Porzana Inc.) prior to release.

All fieldwork was conducted following guidelines for the safe and humane handling of bats published by the American Society of Mammalogists (Sikes and Animal Care and Use Committee of the American Society of Mammalogists 2016) and was approved by the Institutional Animal Care and Use Committees of the University of Georgia (A2014 04‐016‐Y3‐A5), American Museum of Natural History (AMNHIACUC‐20170403, AMNHIACUC‐20180123, AMNHIACUC‐20190129) and University of Oklahoma (2022‐0197). Fieldwork and sampling were authorised by the Belize Forest Department under permits CD/60/3/15(21), WL/1/1/16(17), WL/2/1/17(16), WL/2/1/17(19), WL/2/1/18(16), FD/WL/1/19(06), FD/WL/1/19(09) and FD/WL/1/21(12).

### 2.3 Tree Cover Quantification

To test our third hypothesis, we quantified the tree cover in the region during each year of our study. Tree cover data were not available for 2015 and 2016 and were therefore excluded from the following quantifications. Because of difficulties associated with differentiating between the effects of habitat loss and habitat fragmentation (Laurance 2008; Didham et al. 2012), we used the change in tree cover in the matrix between and surrounding the LAR and KK over time as a proxy for habitat fragmentation. Therefore, our quantifications indicate changes in land cover over time associated with habitat fragmentation as opposed to fragmentation per se. We quantified land cover change between years with ArcGIS Pro version 3.0.3 (Esri Inc.) using data from the Sentinel‐2 10 m Land Use/Land Cover Time Series produced by Impact Observatory, Microsoft and Esri (Karra et al. 2021). We cropped the map to include only land cover data within 10 km from the midpoint between the LAR and KK to encompass an estimated five km home range of Neotropical bats, including vampire bats, for each site (Figure S1) (Trajano 1996; Fenton et al. 2000; Fleming et al. 1972; Becker et al. 2021). We masked the extents of the LAR and KK to focus analyses on the matrix. Individual binary layers were then created for each year (2017–2022), in which pixels belonging to the ‘Trees’ classification were assigned a 1, while pixels belonging to all other classifications were assigned a 0. We quantified the change in tree cover from one year to the next for sequential pairs of years by subtracting the binary layer extent from each pair of successive years (e.g., 2018–2017, 2019–2018). The number of cells with a value of 1 and the number of cells with a value of −1 in the resulting layer were each multiplied by the pixel size of the map (100 m^2^) and converted to square kilometres to determine the area gained and lost, respectively. The area lost was subtracted from the area gained to determine the net change in area per year. We calculated the percent change in forest cover per year by dividing the net change in area by the total area in the first year of each pair of years.

### 2.4 Pathogen Diagnostics and Genotyping

Genomic DNA was extracted from 431 blood samples (319 from the LAR and 112 from KK) using Qiagen QIAamp DNA Investigator Kits. Extracted DNA was screened for *Bartonella* spp. through nested PCR amplification of the partial citrate synthase (*gltA*) gene (Norman et al. 1995; Birtles and Raoult 1996). All PCRs used blank FTA card punches and ultrapure water as extraction and negative controls, respectively. We did not include positive controls of *Bartonella* spp. due to cross‐contamination risks from nested PCR. Amplicons of expected size (~300 bp) were identified by gel electrophoresis using a 2% agarose gel, and PCR was repeated for samples included in gels without any amplicons to ensure assay validity in the absence of a positive control. Amplicons were cleaned using Zymo DNA Clean and Concentrator‐5, Zymoclean Gel DNA Recovery Kits, or Applied Biosystems ExoSAP‐IT PCR Product Cleanup Reagent. Cleaned amplicons were Sanger sequenced at the Georgia Genomics Facility (2015 and 2016 samples) and the North Carolina State University Genomics Sciences Laboratory (2017–2019, 2021, and 2022 samples). DNA extracts were also screened for *Mycoplasma* spp. through PCR amplification of the partial 16S ribosomal RNA (rRNA) gene (Volokhov et al. 2011). For a subset of 16S rRNA–positive samples, we also attempted to amplify the partial 23S ribosomal RNA (rRNA) gene and/or the RNA polymerase beta subunit (*rpoB*) gene to build a broader library of these genes for hemoplasmas (Table S1; Volokhov et al. 2011; Becker, Dyer, Lock, Olbrys, et al. 2025). ‘*Candidatus* Mycoplasma haematozalophi’ DNA was used as a positive control. Hemoplasma amplicons were Sanger sequenced at Psomagen.

Quality control of *Bartonella* sequences was performed by manually trimming and editing in Geneious version 2023.2.1. Hemoplasma 16S rRNA sequences were screened for chimeric sequences using DECIPHER (Wright et al. 2012) and UCHIME (Edgar et al. 2011). Related sequences were identified using NCBI BLASTn, and the top hits were aligned with our sequences and reference sequences using MUSCLE, also in Geneious. Phylogenetic analysis was performed in NGPhylogeny.fr (Lemoine et al. 2019) using maximum likelihood with smart model selection (PhyML + SMS). Sequences were assigned to genotypes based on phylogenetic similarity using previously established cutoffs for the *gltA* gene for bartonellae (96%; La Scola et al. 2003; Becker, Bergner, et al. 2018) and the 16S rRNA gene for hemoplasmas (98.5%; Becker, Speer, et al. 2020). Data from 2015 and 2016 were published previously (Volokhov et al. 2017; Becker, Bergner, et al. 2018), as was a subset of vampire bat data focused only on the LAR from 2017, 2018, and 2019 (DeAnglis et al. 2024). We deposited 199 new sequences to GenBank (*Bartonella* accession numbers PQ758803–PQ759000, PV067250; see Table S1 for hemoplasma accession numbers). Because we amplified multiple hemoplasma genes for a subset of individual bats, we also used these multi‐locus data here to propose novel *‘Candidatus’* species when the same genotype was identified in at least two samples using 16S rRNA and at least one other marker (i.e., 16S rRNA and 23S rRNA; or 16S rRNA, 23S rRNA and *rpoB*) (Volokhov et al. 2012, 2023).

### 2.5 Statistical Analysis

We used the *prevalence* package (Devleesschauwer et al. 2022) in R version 4.2.1 (R Core Team 2022) to estimate site‐ and year‐specific infection prevalence per pathogen including corresponding 95% confidence intervals. We then used a cross‐correlation analysis of the prevalence time series per site to quantify the synchrony in infection dynamics (Shumway and Stoffer 2000). Next, we used generalised linear mixed models (GLMMs) with the *lme4* package (Bates et al. 2015) to test our three hypotheses about habitat fragmentation and spatiotemporal changes in infection dynamics. We included bat identification number (hereafter ‘bat ID’) as a random effect in order to account for recaptured bats that were sampled multiple times across the study period (*n* = 59 individuals). To test our first and second hypotheses, we fit an initial suite of four GLMMs separately for bartonellae and for hemoplasmas to first test if the effect of site (the LAR and KK) varied with year to influence the likelihood of infection while also testing additional predictors such as age class, sex, and reproductive status. We then used four similar GLMMs to test if regional forest loss per year or site‐specific factors influenced the likelihood of infection; year‐specific tree cover was used as a fixed effect instead of year to test our third hypothesis. As land cover data were only available from 2017 onwards, data from bats captured in 2015 and 2016 were excluded from this analysis (*n* = 358). All GLMMs used a binomial distribution, maximum likelihood estimation and the nlminb optimiser. For each pathogen, we used corrected Akaike information criterion (AICc) to compare each four‐model suite that considered additive and interactive effects of site, year, or tree cover and individual‐level covariates using the *MuMIn* package (Burnham and Anderson 2002). We computed Akaike weights (*w*_*i*_) within each model suite and considered models within two ΔAICc of the top model to be competitive. We obtained estimated marginal means of statistically significant predictors using the *emmeans* package, adjusting for the inflated false discovery rate for post hoc comparisons (Benjamini and Hochberg 1995; Lenth 2022). We also calculated marginal and conditional *R* ^2^ (*R* ^2^_*m*_ and *R* ^2^_*c*_) using the *performance* package (Lüdecke et al. 2021).

To assess pathogen diversity over time, we used a Chi‐squared test to compare the frequencies of each *Bartonella* and hemoplasma genotype across and within years. Low recapture rates (16.3%) precluded repeated‐measures analysis. We calculated frequencies for both sites pooled together for each year since there were no samples for KK in 2021 and only three positive samples for KK in 2022. For any genotype with at least 10 observations, we also tested effects of site, year, and tree cover using the same modelling approach used for overall infection status above. We created new binary infection status variables for each genotype for bats only infected with bartonellae or hemoplasmas, respectively. We then used AICc to compare models within each suite of GLMMs. For simplicity, bats identified as infected with more than one genotype were classified according to the genotype most clearly distinguishable from the sequence chromatogram.

Lastly, we used separate general linear models (GLMs) with a Poisson distribution per pathogen to determine if the total number of genotypes observed infecting a recaptured individual bat across the study duration was influenced by sex, number of captures, minimum age, and whether a bat switched from subadult to adult during its capture history. We also used binomial GLMs to determine if the same fixed effects influenced the likelihood a recaptured bat would switch infection status or genotype. Variables used for all models (GLMMs and GLMs) showed no evidence of collinearity through small variance inflation factors (VIF < 2.2). For each pathogen, we estimated approximate infectious periods (mean and range) using individual recapture histories and the time between the first and last year of positivity.

## 3 Results

**FIGURE 2:**
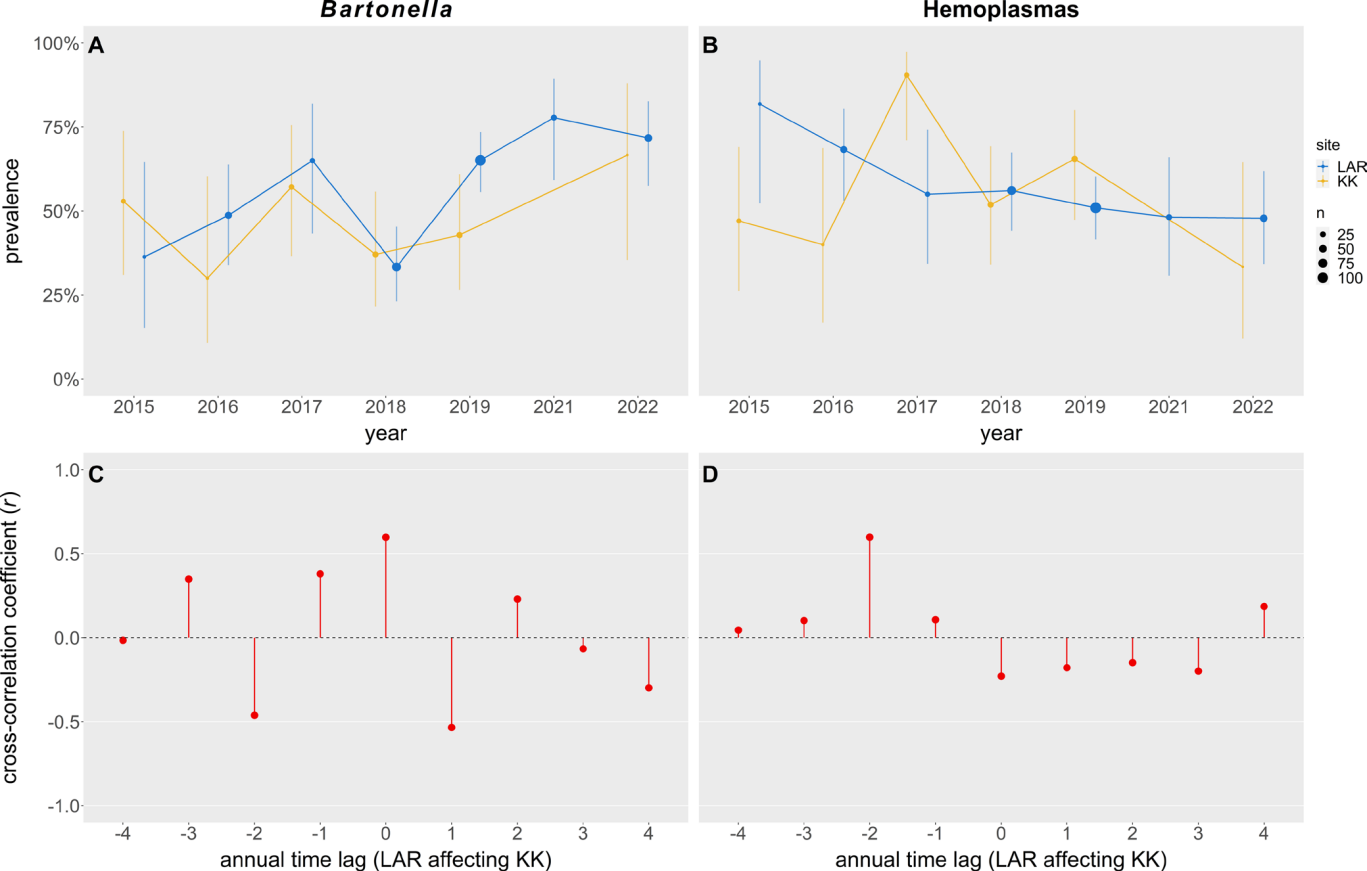
*Bartonella* (A) and hemoplasma (B) prevalence by year in the LAR (blue) and KK (yellow). Point estimates are shown with 95% confidence intervals (Wilson interval) and are dodged to reduce overlap. Cross‐correlation analysis of *Bartonella* (C) and hemoplasma (D) prevalence in the LAR affecting that in KK.

Across all years, 361 unique bats were sampled. A total of 233 blood samples tested positive for *Bartonella* infections out of 427 total blood samples tested, and 241 blood samples tested positive for hemoplasma infections out of 430 total blood samples tested. Throughout the seven‐year study period, *Bartonella* prevalence ranged from 36.4% (CI = 0.15–0.65) to 77.7% (CI = 0.59–0.89) in the LAR and 30.0% (CI = 0.11–0.60) to 66.7% (CI = 0.35–0.88) in KK. At the same time, hemoplasma prevalence ranged from 47.8% (CI = 0.34–0.62) to 81.8% (CI = 0.52–0.95) in the LAR and 33.3% (CI = 0.12–0.65) to 90.5% (CI = 0.71–0.97) in KK (Figure 2A,B). Cross‐correlation analysis suggested high synchrony in prevalence across annual lags in *Bartonella* infections, while we only observed high synchrony in prevalence at the negative two‐year lag for hemoplasma infections, with weak synchrony in prevalence at all other annual lags (Figure 2C,D).

The forest cover within the 10 km matrix around the centroid of the LAR and KK was reduced by 23.8% from 2017 to 2022. Most forest loss occurred within the first two years of the study, with an 8.2% decrease from 9.7 km^2^ in 2017 to 8.9 km^2^ in 2018, then another 14.5% decrease to 7.6 km^2^ in 2019. Forest cover then remained relatively stable through the end of the study, rising briefly by 3.6% to 7.8 km^2^ in 2020 before falling again by 4% to 7.5 km^2^ in 2021 and by another 2.3% to 7.4 km^2^ in 2022 (Figure S2).

### 3.1 Spatiotemporal Predictors of *Bartonella* Infection

**FIGURE 3:**
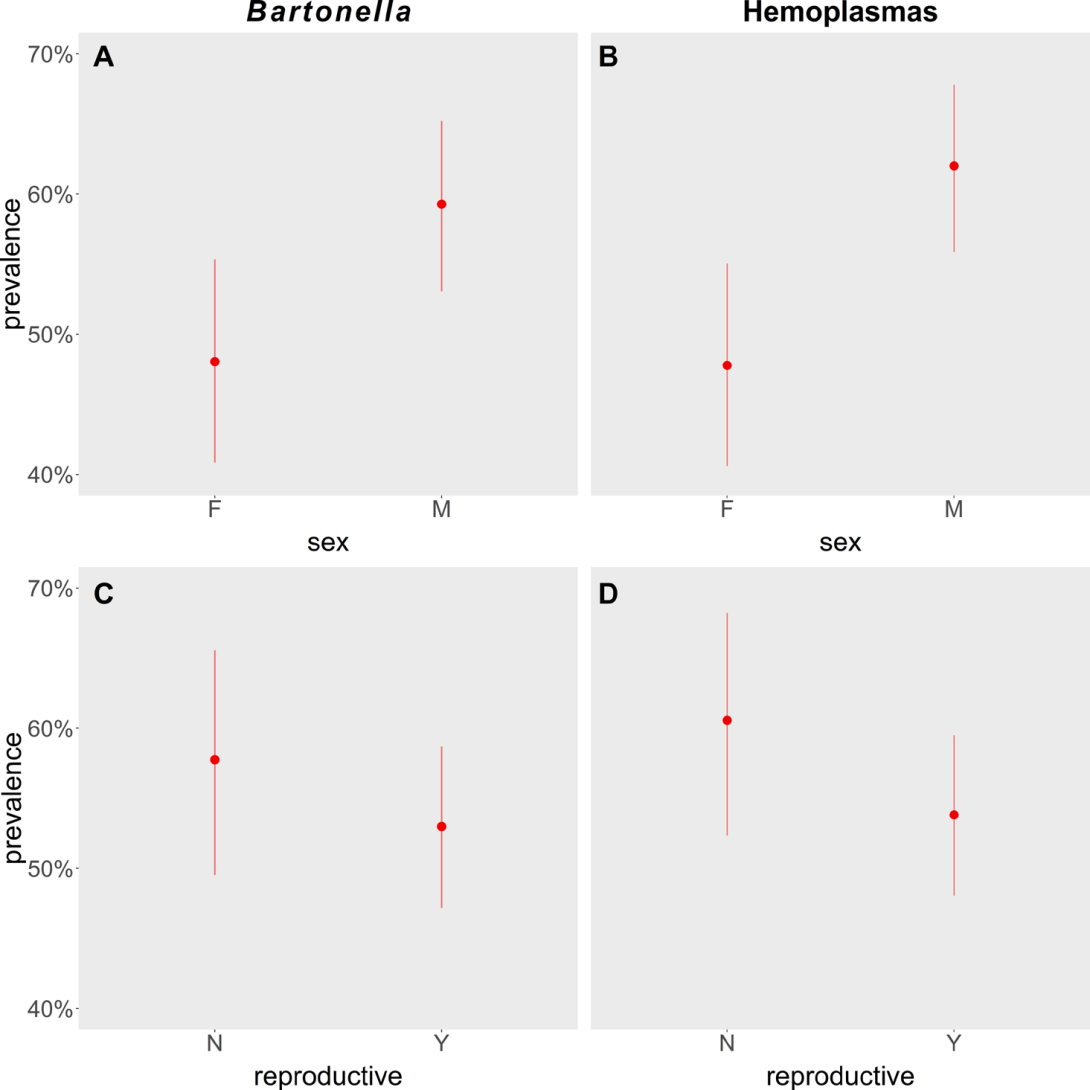
*Bartonella* (A, C) and hemoplasma (B, D) prevalence by sex and reproductive status. Point estimates are shown with 95% confidence intervals (Wilson interval).

Among four GLMMs predicting *Bartonella* infection status across all years (2015–2022), the most competitive model included additive effects of site, year, sex, reproductive status, and age class and no interactive effects (Table S2). Infection was best predicted by year (Table S3), with bats having a significantly different likelihood of infection across pairwise year comparisons. While odds ratios for most post hoc comparisons between years were generally similar and non‐significant (OR = 0.022–2.82, *p* ≥ 0.06), bats were significantly more likely to be infected in 2021 (OR = 0.19, *p* = 0.02) and 2022 (OR = 0.26; *p* = 0.02) compared to 2016 but less likely in 2018 compared to 2019 (OR = 0.35, *p* < 0.01), 2021 (OR = 0.16, *p* < 0.01) and 2022 (OR = 0.22, *p* < 0.01) (Figure 2A). Sex and reproductive status also predicted *Bartonella* infection, with males having a significantly higher likelihood of infection than females (OR = 2.30, *p* < 0.01; Figure 3A) and reproductive bats having a lower likelihood of infection than nonreproductive bats (OR = 0.55, *p* = 0.05; Figure 3C). There was no significant effect of site or age class (Tables S3 and S4). The fixed effects accounted for 14% of the variance in infection status (*R* ^2^_*m*_ = 0.14) while bat ID accounted for 7% (*R* ^2^_*c*_ = 0.21), indicating that recaptured bats (*n* = 59) had little effect on the model predictions.

### 3.2 Effect of Tree Cover on *Bartonella* Infection

**FIGURE 4:**
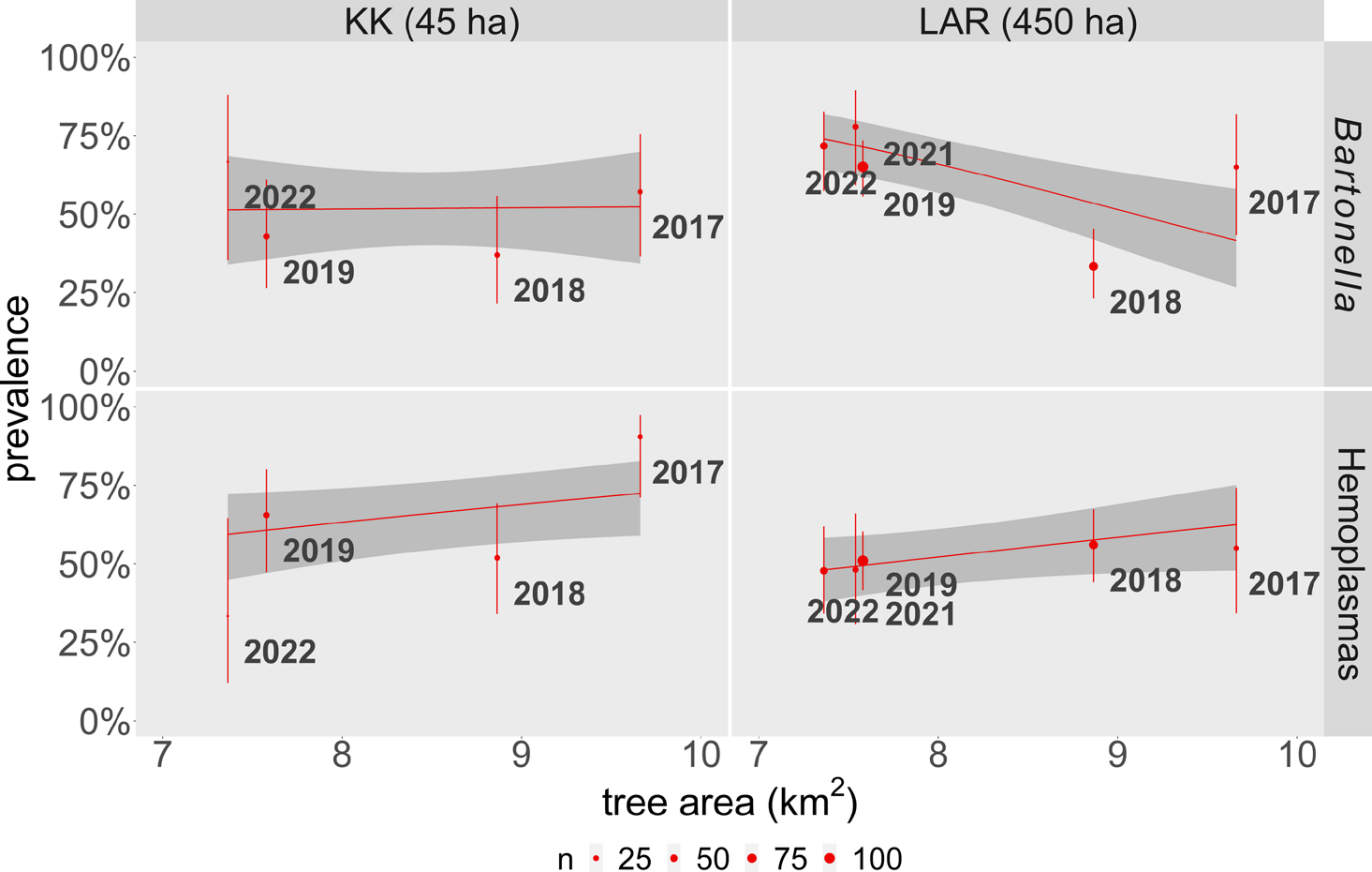
*Bartonella* and hemoplasma infection prevalence by tree cover in each site from 2017 to 2022. Point estimates are shown with 95% confidence intervals (Wilson interval). Overlaid are the 95% confidence bands and fitted values from the respective GLMMs.

For the subset of our *Bartonella* data that included tree cover (2017–2022), the GLMM with an interaction between site and the area of tree cover in the matrix and additive effects of sex, reproductive status, and age class was superior (Table S2). Infection was best predicted by the interaction between site and tree cover and by sex (Table S5). While the area of tree cover in the matrix had no effect on *Bartonella* infection in KK (OR = 1.02, *p* = 0.95), we found that decreased tree cover was associated with a higher infection likelihood in the LAR (OR = 1.83, *p* < 0.001; Figure 4). As in the GLMMs fit to the full dataset, males were more likely to be infected by females (OR = 2.38, *p* < 0.01). There was no effect of age class or reproductive status (Tables S5 and S6). The fixed effects accounted for 11% of the variance in the data (*R* ^2^_*m*_ = 0.11), with no additional contribution from bat ID (*R* ^2^_*c*_ = 0.11).

### 3.3 *Bartonella* Genotype Diversity (2015–2022)

We identified 16 different *Bartonella* genotypes in this study (Figure S3). Nine of these (DR1, 2, 4–6, 8–11) had been identified in our previous study of vampire bats (Becker, Bergner, et al. 2018) and we had previously identified one genotype (TB1) in Mexican free‐tailed bats ( *Tadarida brasiliensis* ) in Oklahoma (Becker, Dyer, Lock, Olbrys, et al. 2025). The remaining six genotypes (DR12–17) were novel. The genotypes identified were highly similar to reference sequences from bat and bat fly species but not to reference sequences isolated from humans, domestic animals, or other wildlife. The two sequences assigned to DR12 (GenBank accessions PQ758896 and PQ758863) were nearly identical (> 99%) to sequences previously found in a streblid bat fly (*Megistopoda proxima*) found on *Sturnira parvidens* from Costa Rica (MH234363). Meanwhile, five sequences (PQ758813, PQ758901, PQ758909, PQ758910, PQ758950) were assigned to DR13 and were 99%–100% similar to other bartonellae previously found in vampire bats from Brazil (PP715670), Mexico (MF467787) and Guatemala (MN529504). Only one sequence made up each of the DR14, DR15, DR16, and DR17 genotypes. The DR14 sequence (PQ758852) was 96.2% similar to a *Bartonella* spp. from *Myotis nigricans* from Guatemala (MN529480). DR15 (PQ758988) was only 88.1% similar to *Bartonella* spp. from a bat fly in China (species not specified; MZ208693). DR16 (PQ758964) was 93.7% similar to a *Bartonella* spp. from *Carollia perspicillata* from Costa Rica (MH234350). Lastly, DR17 (PQ758949) was 93.1% similar to a *Bartonella* spp. from *Glossophaga mutica* (formerly *G. soricina* ) from Costa Rica (MH234356).

As most of the genotypes were observed < 10 times each across the entire study period, only those with at least 10 observations were included in subsequent analyses: DR1 (*n* = 25), DR2 (*n* = 21), DR8 (*n* = 32), DR9 (*n* = 31), and DR11 (*n* = 56), all of which were identified previously (Becker, Bergner, et al. 2018). The frequencies of DR8 (*χ* ^2^ = 17.4, *p* < 0.01), DR9 (*χ* ^2^ = 26.1, *p* < 0.01), and DR11 (*χ* ^2^ = 56.5, *p* < 0.01) all varied significantly across years, but those of DR1 (*χ* ^2^ = 7.40, *p* = 0.19) and DR2 (*χ* ^2^ = 6.71, *p* = 0.24) did not. These five dominant genotypes oscillated through periods of equilibrium and periods of dominance by one or more genotypes. The most recent years of sampling saw no significant differences in genotype frequency during 2021 (*χ* ^2^ = 4.47, *p* = 0.35) or 2022 (*χ* ^2^ = 1.67, *p* = 0.80). The same was true for 2015 (*χ* ^2^ = 0.67, *p* = 0.96) and 2017 (*χ* ^2^ = 4.00, *p* = 0.41), with periods of significant differences in *Bartonella* genotype frequencies in 2018 (*χ* ^2^ = 12.4, *p* = 0.01) and 2019 (*χ* ^2^ = 24.5, *p* < 0.01) when DR11 was 22% and 15% more prevalent than the next most prevalent genotype, respectively (Figure S4). Only DR8 was detected in 2016, preventing statistical comparison but demonstrating a period of dominance by DR8 over others.

### 3.4 Spatiotemporal Predictors of *Bartonella* Genotypes

The GLMM that best predicted infection with each respective genotype among *Bartonella*‐infected bats included the additive effects of site, year, sex, reproductive, status, and age class but no interactive effects (Table S7). However, significant predictors were not identified for any of the five dominant genotypes (Tables S8 and S13). The fixed effects accounted for 55%–57% of the variance in the data for DR1 (*R* ^2^_*m*_ = 0.56), DR2 (*R* ^2^_*m*_ = 0.54), DR9 (*R* ^2^_*m*_ = 0.57), and DR11 (*R* ^2^_*m*_ = 0.55), respectively, while they only accounted for 10% of the variance in the data for DR8 (*R* ^2^_*m*_ = 0.10). No additional variance was attributed to bat ID (DR1 *R* ^2^_*c*_ = 0.56; DR2 *R* ^2^_*c*_ = 0.54; DR8 *R* ^2^_*c*_ = 0.10; DR9 *R* ^2^_*c*_ = 0.57; DR11 *R* ^2^_*c*_ = 0.55).

### 3.5 Effect of Tree Cover on *Bartonella* Genotypes

**FIGURE 5:**
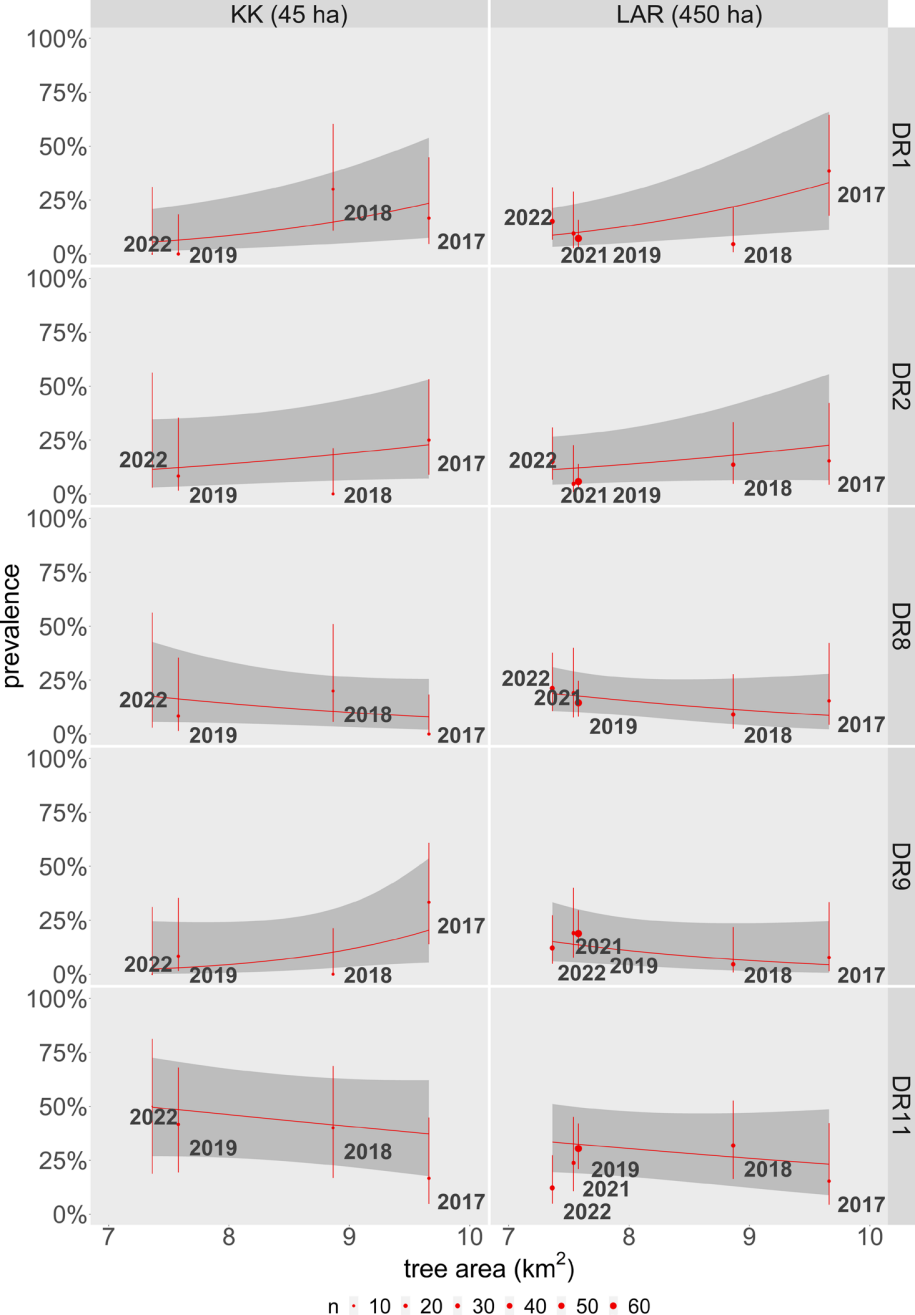
Prevalence (point estimates and 95% confidence intervals [Wilson interval]) of *Bartonella* genotype infections by tree cover from 2017 to 2022. Overlaid are the 95% confidence bands and fitted values from the respective GLMMs.

The GLMM that best predicted infections with DR1, DR2, DR8, and DR11 among *Bartonella*‐infected bats included the additive effects of site, tree area, sex, reproductive status, and age class but no interactive effects (Table S14). The area of tree cover in the matrix was the only significant predictor of DR1 infection (Tables S15 and S16), in which decreasing tree cover was associated with a decreased likelihood of infection (OR = 0.49, *p* = 0.01; Figure 5). We did not identify significant predictors of DR2, DR8, or DR11 infections (Tables S15, S17, S18, and S20). The model that best predicted DR9 infections included the interaction between site and tree area as well as additive effects of sex, reproductive status, and age class (Table S14). The interaction between site and tree cover was a significant predictor of DR9 infection (Tables S15 and S19), but we did not identify post hoc differences in infection likelihood between sites by tree cover (OR_KK_ = 2.80, *p* = 0.12; OR_LAR_ = 0.55, *p* = 0.16; Figure 5). The fixed effects included in the models accounted for 10%, 3%, 9%, 15%, and 3% of the variance in the data for DR1 (*R* ^2^_*m*_ = 0.10), DR2 (*R* ^2^_*m*_ = 0.03), DR8 (*R* ^2^_*m*_ = 0.09), DR9 (*R* ^2^_*m*_ = 0.15), and DR11 (*R* ^2^_*m*_ = 0.03), respectively. No additional variance was attributed to bat ID for DR1 (*R* ^2^_*c*_ = 0.10), DR2 (*R* ^2^_*c*_ = 0.03), DR8 (*R* ^2^_*c*_ = 0.09), DR9 (*R* ^2^_*c*_ = 0.15), or DR11 (*R* ^2^_*c*_ = 0.03).

### 3.6 *Bartonella* Infection Status and Genotype Switching Among Recaptured Bats

**FIGURE 6:**
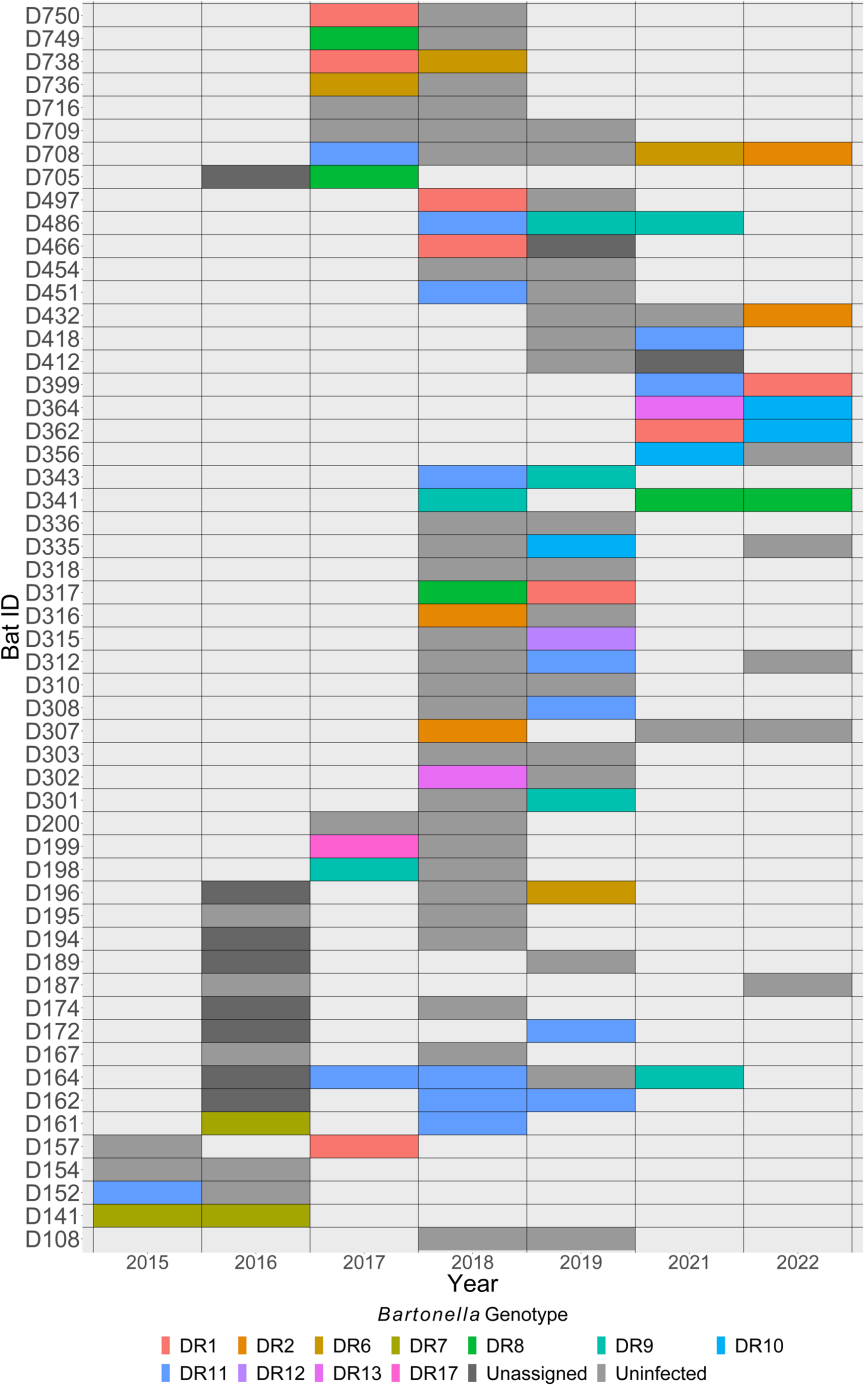
*Bartonella* infection status and genotypes over time in bats recaptured across multiple years.

Across the entire study period, 59 bats were recaptured at least once (145 total captures). Recaptured bats were captured an average of 2.42 times (range = 2–5 captures per bat). Of the recaptured bats, 19 were never found to be infected with *Bartonella*. Twenty‐six bats switched infection status during our study: 13 individuals switched from positive to negative, seven switched from negative to positive, four switched from positive to negative and then back to positive, and two switched from negative to positive and then back to negative. Eleven bats with multiple years of *Bartonella* infections switched genotypes. Five bats were infected with the same genotype two years in a row, with no recaptured bats maintaining the same genotype over a longer period of time (mean infectious period = 1 year). While two different genotypes were detected sequentially in 10 of the 40 recaptured bats with *Bartonella* infections, only one recaptured bat harboured infections with three different genotypes across the study period and only one genotype was detected in 29 of the recaptured bats (Figure 6).

The number of captures per bat was the only predictor of the number of genogroups identified from a single individual and whether infected bats switched between genogroups across capture events (Tables S21, S22, and S24). Bats with a higher number of capture events were also more likely to host a higher number of *Bartonella* genotypes across the study period (OR = 1.72, *p* = 0.02; Figure S5). Additionally, bats with more capture events were more likely to switch between *Bartonella* genotypes (OR = 7.26, *p* = 0.02). We did not identify any predictors of *Bartonella* infection status switching (Tables S21 and S23).

### 3.7 Spatiotemporal Predictors of Hemoplasma Infection

Among four GLMMs predicting hemoplasma infection status across all years (2015–2022), the most competitive model included the interaction between site and year as well as additive effects of sex, reproductive status, and age class (Table S2). Infection was best predicted by sex, the interaction between site and year, and reproductive status (Table S3). While we did not identify any post hoc differences in infection likelihood between sites by year (OR = 0.07–24.2, *p* = 0.1–1.0), males were more likely to be infected than females (OR = 2.38, *p* < 0.01; Figure 3B) and non‐reproductive bats were more likely to be infected than reproductive bats (OR = 0.54, *p* = 0.04; Figure 3D). There was no effect of age class on hemoplasma infection (Tables S3 and S4). The fixed effects accounted for 15% of the variance in infection status (*R* ^2^_*m*_ = 0.15), with bat ID explaining an additional 9% of the variance (*R* ^2^_*c*_ = 0.24).

### 3.8 Effect of Tree Cover on Hemoplasma Infection

For the subset of our hemoplasma data that included tree cover (2017–2022), the GLMM with additive effects of site, tree cover, sex, reproductive status, and age class, but no interactive effects, was superior (Table S2). Infection was best predicted by sex and reproductive status (Table S5). As in our analyses with the full dataset, males were more likely to be infected than females (OR = 2.02, *p* ≤ 0.01), while non‐reproductive bats had higher likelihoods of infection compared to reproductive bats (OR = 0.47, *p* ≤ 0.01). There was no significant effect of site, tree cover, or age class on infection status (Tables S5 and S6). The fixed effects accounted for 8% of the variance in infection status (*R* ^2^_*m*_ = 0.08). No additional variance was attributed to bat ID (*R* ^2^_*c*_ = 0.08).

### 3.9 Hemoplasma Genotypic Diversity (2015–2022)

As suggested by our earlier work (Volokhov et al. 2017; Becker, Speer, et al. 2020; DeAnglis et al. 2024), three genotypes (VB1, VB2, VB3) comprised the majority of hemoplasma infections (240 of 242 total hemoplasma‐positive bats). In addition, one bat (OQ385174) was infected with a potentially novel hemoplasma genotype with a 16S rRNA gene sequence 99% similar to that of genotype MR1 (MH245174), which was previously found in *Molossus nigricans* and *Molossus alvarezi* (Becker, Speer, et al. 2020; Volokhov et al. 2023). Another (OQ546513) was identified as belonging to genotype EF1 (GenBank accession MH245131), which typically infects *Neoeptesicus furinalis* (formerly *Eptesicus furinalis)* but has also been found in *Glossophaga mutica* (formerly *G. soricina* ) and *Saccopteryx bilineata* (Becker, Speer, et al. 2020; Volokhov et al. 2023; Figure S6). As previously reported, a third 16S rRNA sequence (KY932724) was 98% similar to *Mycoplasma moatsii* (NR_025186), a non‐hemotropic species and therefore not considered to be a hemoplasma (Volokhov et al. 2017). These genotypes did not share a high degree of genetic similarity to reference sequences from humans, domestic animals, other wildlife, or potential arthropod vectors (Figure S6). Across all bats in our study, 17 were coinfected with multiple hemoplasma genotypes and were classified according to the dominant genotype. Only infections belonging to the dominant genotypes were included in subsequent analyses: VB1 (*n* = 106), VB2 (*n* = 85) and VB3 (*n* = 49). Amplification of paired partial 23S rRNA and/or *rpoB* genes for samples belonging to these 16S rRNA genotypes (see Table S1) suggested at least two novel ‘*Candidatus*’ hemoplasma species circulate in *Desmodus rotundus* . Based on 99.9% identity of three 23S rRNA sequences (OQ518936, OQ518940, OQ518941) and 99.9%–100% identity of paired 16S rRNA sequences included in the VB1 genotype (MH245176, OQ546521, OQ546522), first detected in the LAR, we propose the name ‘*Candidatus* Mycoplasma lamanaiense’ sp. nov., (*la.ma.na.i.en'se*. N.L. neut. adj. *lamanaiense*, pertaining to Lamanai, the archaeological reserve in Belize where the organism was discovered); paired *rpoB* sequences in two of these three bats also showed 100% identity (OQ554324, OQ554325), further confirming this proposed hemoplasma species. Similarly, given 99.9%–100% identity among three 23S rRNA sequences (OQ518937, OQ518938, OQ518946) and 99.1%–99.4% identity of paired 16S rRNA sequences included in the VB2 genotype (OQ385162, OQ546569, OQ546517), first detected in KK, we propose the name ‘*Candidatus* Mycoplasma kakabishense’ sp. nov., (*ka.ka.bish.en'se*. N.L. neut. adj. *kakabishense*, pertaining to Ka'Kabish, the archaeological site in Belize where the organism was detected) nov.

The frequency of all three of the main hemoplasma genotypes varied significantly among years (VB1 *χ* ^2^ = 22.2, *p* < 0.01; VB2 *χ* ^2^ = 64.5, *p* < 0.01; VB3 *χ* ^2^ = 26, *p* < 0.01). The frequency of VB1 decreased from 2017 to 2019 while that of VB2 increased during the same timespan. The frequency of VB3 has gradually increased throughout the study period, and the three genotypes have reached an equilibrium in the most recent years of sampling, with no significant differences in frequency during 2021 (*χ* ^2^ = 1.08, *p* = 0.58) or 2022 (*χ* ^2^ = 0.75, *p* = 0.69). Genotype frequencies were also nonsignificant during 2015 (*χ* ^2^ = 5.76, *p* = 0.06) and 2018 (*χ* ^2^ = 0.82, *p* = 0.66), but varied significantly during 2016 (*χ* ^2^ = 22.6, *p* < 0.01), 2017 (*χ* ^2^ = 13.6, *p* < 0.01), and 2019 (*χ* ^2^ = 10.1, *p* < 0.01) (Figure S7).

### 3.10 Spatiotemporal Predictors of Hemoplasma Genotypes

Among the four models predicting infection with each respective hemoplasma genotype among infected bats across all years (2015–2022), the GLMM that best predicted infection across genotypes included the additive effects of site, year, sex, reproductive status, and age class but no interactive effects (Table S25). VB1 infection was best predicted by year (Table S26). VB1 infection was significantly more likely in 2016 compared to 2018 (OR = 6.71, *p* < 0.01), 2019 (OR = 6.18, *p* < 0.01), and 2022 (OR = 4.65, *p* = 0.04) and significantly more likely in 2017 compared to 2018 (OR = 5.07, *p* = 0.01) and 2019 (OR = 4.67, *p* = 0.01) (Figure S7). There were no effects of site, sex, age class, or reproductive status on VB1 infection (Tables S26, S27). The fixed effects accounted for 16% of the variance in the data (*R* ^2^_*m*_ = 0.16), while no additional variance was attributed to bat ID (*R* ^2^_*c*_ = 0.16).

Infection with VB2 among hemoplasma‐infected bats was best predicted by year (Table S26). VB2 infection was significantly less likely in 2016 (OR = 0.23, *p* = 0.05) and 2017 (OR = 0.16, *p* = 0.03) compared to 2019 (Figure S7). There were no effects of site, sex, age class, or reproductive status (Tables S26 and S27). The fixed effects accounted for 18% of the variance in the data (*R* ^2^_*m*_ = 0.18). No additional variance was attributed to bat ID (*R* ^2^_*c*_ = 0.18).

We did not identify any significant predictors of VB3 infection (Tables S26 and S27). Fixed effects accounted for only 17% of variance (*R* ^2^_*m*_ = 0.17). Bat ID did not explain further variance (*R* ^2^_*c*_ = 0.17).

### 3.11 Effect of Tree Cover on Hemoplasma Genotypes

**FIGURE 7:**
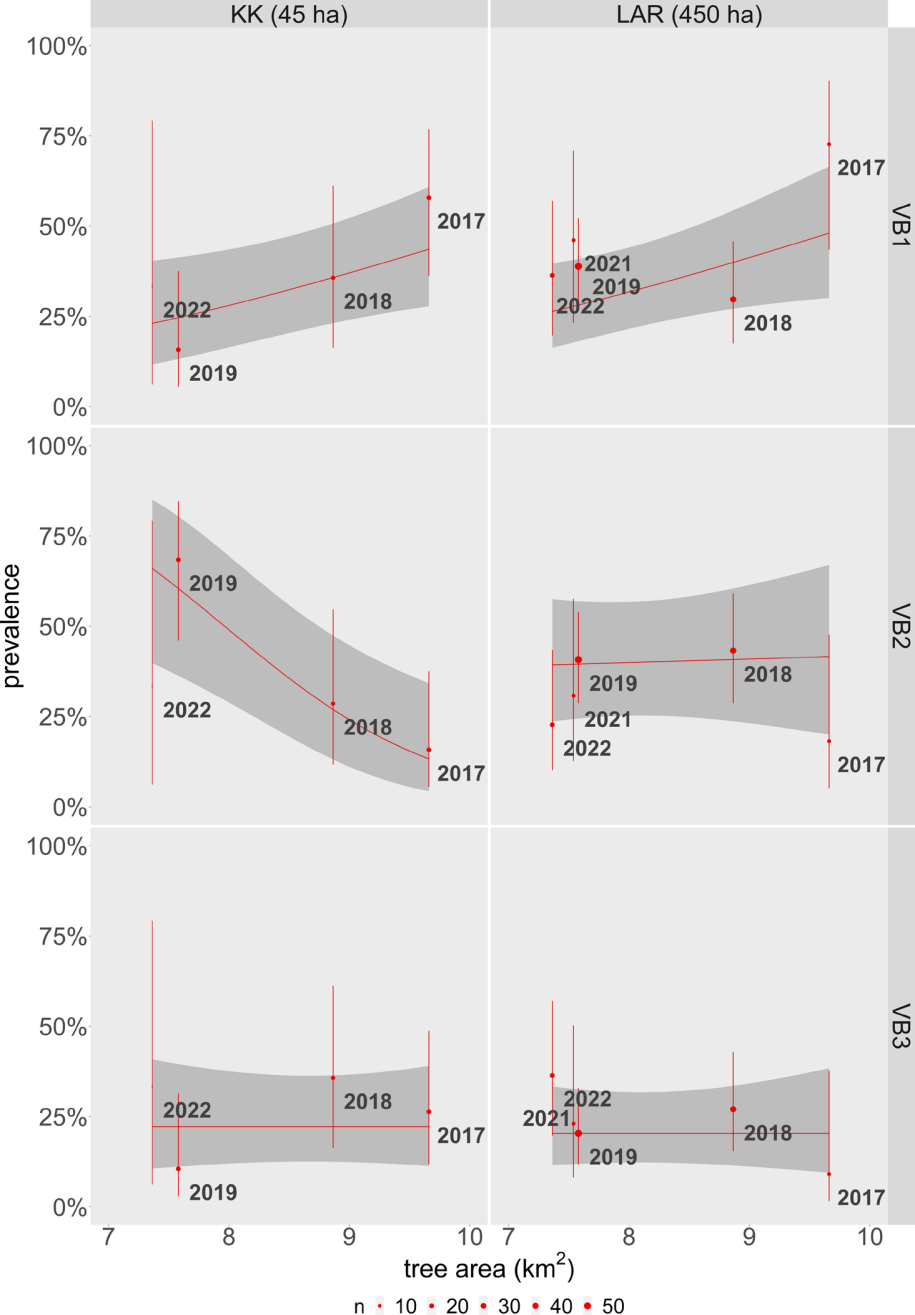
Prevalence of hemoplasma genotype infections by tree cover from 2017 to 2022. Point estimates are shown with 95% confidence intervals (Wilson's interval). Overlaid are the 95% confidence bands and fitted values from the respective GLMMs.

For the subset of hemoplasma‐infected bats for which tree cover data were available (2017–2022), the model that best predicted infection with VB1 included the additive effects of site, tree cover, sex, reproductive status, and age class but no interactive effects (Table S28). VB1 infection was best predicted by tree cover (Table S29), where decreasing tree cover was negatively associated with infection likelihood (OR = 0.66, *p* = 0.03; Figure 7). There was no effect of site, sex, age class, or reproductive status on the odds of VB1 infection (Tables S29 and S30). The fixed effects accounted for 5% of the variance in the data (*R* ^2^_*m*_ = 0.05), with no additional variance attributed to bat ID (*R* ^2^_*c*_ = 0.05).

The model that best predicted infection with VB2 included the interaction between site and tree area along with sex, reproductive status, and age class (Table S28). VB2 infection was best predicted by the interaction between tree area and site (Table S29). Decreasing tree cover was positively associated with VB2 infection in KK (OR = 3.02, *p* < 0.01) but not in the LAR (OR = 1.01, *p* = 0.86; Figure 7). We detected no significant effect of sex, reproductive status, or age class (Tables S29 and S30). The fixed effects accounted for 12% of the variance in infection status (*R* ^2^_*m*_ = 0.12). No additional variance was attributed to bat ID (*R* ^2^_*c*_ = 0.12).

The top model for VB3 among hemoplasma‐infected bats included additive effects of site, tree cover, sex, reproductive status, and age class but no interactive effects (Table S28). However, we identified no significant predictors of VB3 infection (Tables S29 and S30). The fixed effects explained 3% of the variance in the data (*R* ^2^_*m*_ = 0.03), with no additional contribution from bat ID (*R* ^2^_*c*_ = 0.03).

### 3.12 Hemoplasma Infection Status and Genotype Switching Among Recaptured Bats

**FIGURE 8:**
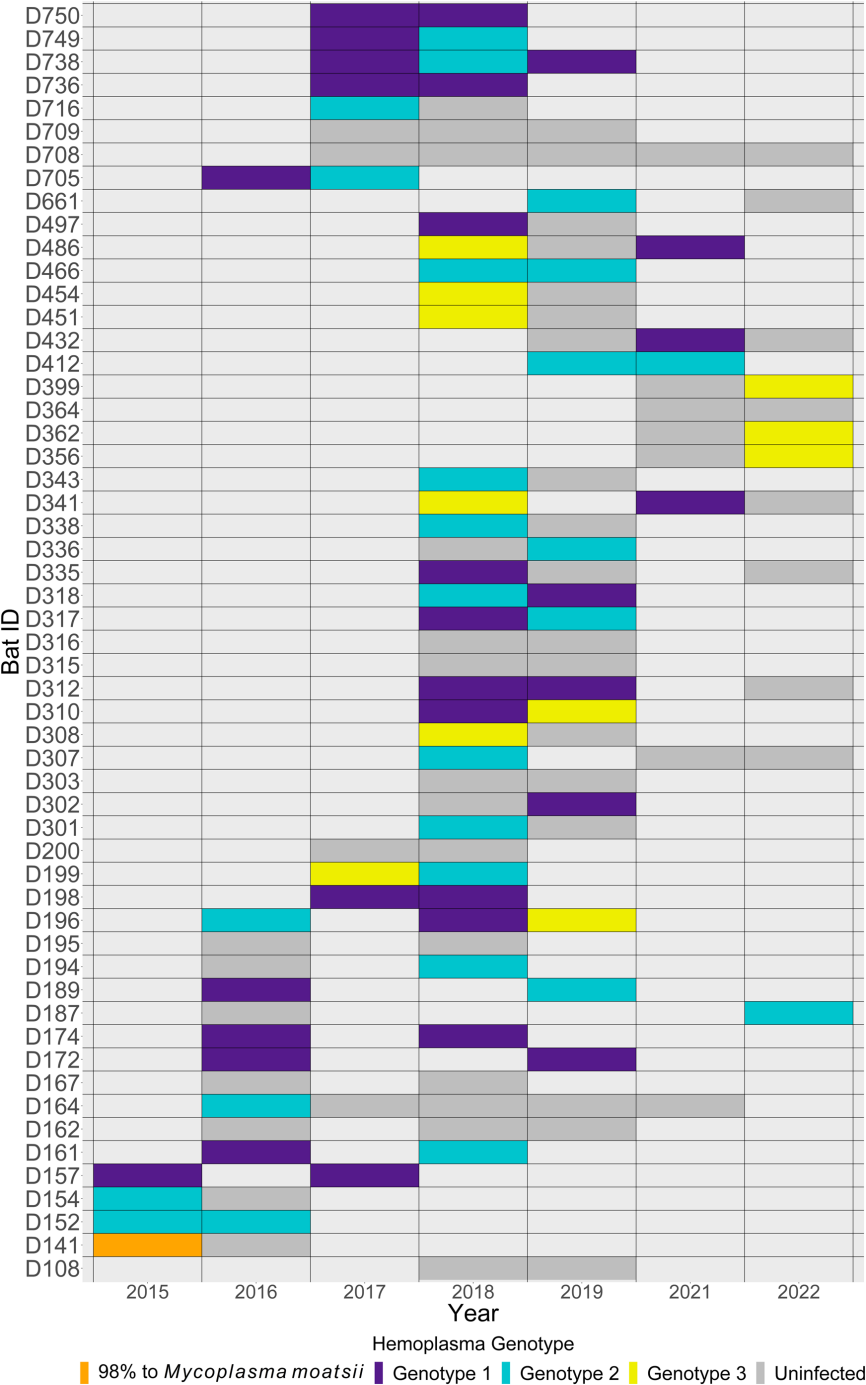
Hemoplasma infection status and genotypes over time in bats recaptured across multiple years.

We detected hemoplasma infections from 48 of the 59 bats recaptured during this study. Twenty‐five bats changed infection statuses, with 14 switching from positive to negative, eight switching from negative to positive, two switching from positive to negative and then back to positive, and one switching from negative to positive and then back to negative. Twelve bats with multiple hemoplasma infections switched between genotypes. Most recaptured bats (*n* = 29, 60%) only had one detected genotype during the study period. Twelve bats were infected with two hemoplasma genotypes at some point during the study period, and none carried more than two genotypes (Figure 8). Ten bats maintained the same genotype across multiple years, with a mean infectious period of 1.4 years (range = 1–3 years). However, we did not identify any predictors of genotype switching, infection status switching, or the number of genotypes identified in a recaptured bat across the study period (Tables S31–S34).

## 4 Discussion

Habitat fragmentation typically has detrimental effects on wildlife, including increased risk of pathogen exposure (Haddad et al. 2015; Plowright et al. 2021; Gibb et al. 2024). However, testing for such effects is difficult, as the effects of habitat fragmentation can take years to manifest (Heckley et al. 2023) and are challenging to distinguish from effects of habitat loss (Laurance 2008; Didham et al. 2012). Here, we show that despite similarities in infection prevalence within our studied vampire bat populations, bartonellae and hemoplasmas varied in their spatiotemporal dynamics within the changing landscape and in their responses to forest loss. Additionally, responses to spatiotemporal and individual‐level factors varied by genotype within both of the two respective pathogens, suggesting that the effects of habitat fragmentation on infectious disease dynamics may not be easily generalisable across or within pathogens. However, as the explanatory power of our models was low, additional studies are needed to confirm these trends and identify their mechanistic drivers.

### 4.1 Bartonellae and Hemoplasmas Differ in Their Spatiotemporal Dynamics

Although this study cannot explicitly define the mechanisms linking forest change with differential infection prevalence by pathogen or genotype, contrasting infection dynamics between bartonellae and hemoplasmas may be due to differences in the relative support for vector‐borne and direct transmission (Willi et al. 2007; Judson et al. 2015; Becker, Bergner, et al. 2018; McKee et al. 2026). While the lack of significant site differences in our spatiotemporal models did not support our first hypothesis that infection risk would be higher in the smaller habitat, our second hypothesis was supported, as vampire bats were more likely to be infected with *Bartonella* in the last three years of our study compared to earlier years (Figure 2A). This may reflect a potential lag in the effects of forest loss that may have both preceded our work and occurred in earlier years of our study (Heckley et al. 2023). However, this possibility is difficult to explore, since land cover data were not available for the first two years of our study. In the future, this challenge could be overcome by utilising land cover data from multiple sources of satellite imagery, as the data used here were derived from Sentinel‐2 images only (Li and Roy 2017; Claverie et al. 2018). Additionally, the difference in infection likelihood across sites in 2021 compared to other years may indicate an effect of seasonality, as 2021 sampling occurred in November instead of our typical sampling during April and May. While travel restrictions due to the COVID‐19 pandemic prevented sample collection during the same season for this particular study, future studies could avoid confounding seasonal effects by sampling at the same time each year. Alternatively, seasonal drivers of infection could be investigated by sampling during both the dry and wet seasons across years (Becker, Dyer, Lock, Fenton, et al. 2025).

Although site did not predict *Bartonella* infection likelihood and site‐switching bats were rare in this dataset (*n* = 1; consistent with previous reports from a broader mark–recapture dataset of these populations; Becker et al. 2021), we observed strong synchrony in *Bartonella* prevalence between sites across multiple annual lags and in different directions (Figure 2C). While site did not predict *Bartonella* infection likelihood, the interaction between site and year was an important predictor of hemoplasma infection. Although post hoc differences in infection likelihood between sites by year were not strong enough to be statistically significant, we did find a high degree of synchrony in infection prevalence between KK and the LAR at the negative two‐year lag (Figure 2D), indicating that hemoplasma infection trends in the LAR tend to lead those in KK by two years. Frequent movement among populations is expected to homogenise infection dynamics and promote synchrony (Becker et al. 2021). Within this system, synchrony among sites may be driven by encounters between vampire bats from different sites during foraging (Greenhall et al. 1971). Forest clearing may not be enough to truly isolate vampire bats in the region due to their affinity for feeding on livestock, which are common in the matrix but not within the LAR or KK. Indeed, previous work has shown that over time, the diets of vampire bats from the LAR and KK have converged, with stable isotope data indicating a shift from a more heterogeneous diet to one primarily consisting of livestock blood (Becker, Czirják, et al. 2018). Explicit tracking of vampire bat movements and contact patterns, such as that facilitated by lightweight GPS dataloggers and proximity loggers, could explicitly assess this hypothesis in future work (Ripperger et al. 2019; Wild et al. 2022).

Much more genetic diversity was observed in vampire bat *Bartonella* compared to hemoplasmas, although this may be due to different phylogenetic similarity thresholds for delineating *Bartonella* (i.e., 96% similarity of the *gltA* gene; La Scola et al. 2003; Becker, Bergner, et al. 2018) and hemoplasma genotypes (i.e., 98.5% similarity of the 16S rRNA gene; Becker, Speer, et al. 2020) as well as our inability to detect coinfections. Three of our main *Bartonella* genotypes (DR8, DR9, and DR11) showed significant differences in frequency among years, suggesting that some genotypes (i.e., DR1 and DR2) may be better established and less influenced by extrinsic factors than others (Figure S4). Spatiotemporal factors did not predict infection likelihood for the main genotypes, despite their frequencies differing significantly during a two‐year period from 2018 to 2019 and in 2016 when only DR8 was detected, indicating that the drivers of these differences may not be spatiotemporal in nature. However, the methods used here to detect (i.e., nested PCR) and genotype (i.e., Sanger sequencing) these infections preclude our ability to detect coinfections. Therefore, these results may represent cycling between dominant genotypes within samples with multiple genotypes present and apparent absences or genotype turnover may instead reflect shifts in relative abundance rather than true gains or losses (Mideo et al. 2008).

While hemoplasma genotype richness was much lower than that seen in *Bartonella*, we observed complex dynamics at the genotype level. In keeping with previous studies of this system (Volokhov et al. 2017; Becker, Speer, et al. 2020; DeAnglis et al. 2024), we identified a total of five genotypes, two of which were only observed once. These latter genotypes were previously observed only in non‐vampire bat species (i.e., *Neoeptesicus furinalis, Saccopteryx bilineata, Glossophaga mutica, Molossus nigricans,* and *Molossus alvarezi* ), suggesting limited but existing possibility for transmission between sympatric and possibly co‐roosting bat species. Additionally, the 17 vampire bats found to be coinfected with multiple hemoplasma genotypes may indicate a robust contact network between individuals that facilitates the transmission of hemoplasmas within the LAR, KK, and the surrounding area. This may also be true for *Bartonella*, as coinfections are common in vampire bats (Bai et al. 2011); however, the use of nested PCR and Sanger sequencing in this study did not enable identifying mixed infections.

All three main hemoplasma genotypes differed in frequency over time but apparently reached equilibrium in the two most recent years of the study. Early decreases in the VB1 genotype mirrored by early increases in the VB2 genotype suggest that competition among genotypes may have resulted in a more balanced distribution in the hemoplasma community within vampire bats in recent years and allowed for the steady rise of VB3 (Mideo et al. 2008). While the infection status of hemoplasmas as a whole was influenced by the interaction between site and year, that of VB1 and VB2 was best predicted by year alone, whereas VB3 likelihood did not vary between years. Differences in infection dynamics between hemoplasmas as a whole and among genotypes suggest that hemoplasma lineages vary in their within‐host dynamics and transmission strategies (Mideo et al. 2008; Handel and Rohani 2015).

### 4.2 Responses to Forest Loss Were Variable Between and Within Pathogens

While site and tree cover both influenced infection likelihoods, both strength and interaction effects differed between *Bartonella* and hemoplasmas. *Bartonella* infection risk was positively associated with decreasing tree cover in the matrix, but only within the LAR (Figure 4), suggesting that tree cover loss in the region as a whole may lead to more *Bartonella* infections in the larger habitat patch but not the smaller habitat patch. The decline in tree cover may be enhancing *Bartonella* transmission by facilitating contacts between bat flies and bats (Mello et al. 2023), by altering physiological stress and immune response (Messina et al. 2018), or by forcing bats that lived in the destroyed forest to move in the LAR. While we expected that habitat fragmentation would increase infection likelihood in KK more so than in the LAR, this was not supported by our data, suggesting that KK may be too small for additional loss in tree cover to affect overall *Bartonella* infection dynamics. Of the five main *Bartonella* genotypes we identified, only one was associated with forest loss (Figure 5). This result again suggests that genotypes likely differ in transmission‐relevant traits (e.g., infectiousness, infectious periods, etc.) that shape how their dynamics respond to habitat fragmentation (Mideo et al. 2008; Handel and Rohani 2015; McCallum et al. 2017).

In contrast with *Bartonella*, we did not find that site, tree cover, or their interaction were associated with the likelihood of hemoplasma infections overall. However, two of the hemoplasma genotypes were significantly associated with forest loss, but with opposing trends and differing importance of site (Figure 7). The variation in responses to forest loss among pathogen genotypes may again indicate contrasting transmission processes between genotypes (Handel and Rohani 2015), which may have been differentially affected by tree cover loss within the matrix. Additionally, as proposed to explain the spatiotemporal trends that we observed, there may be competition between genotypes, in which the decline in prevalence of some genotypes may allow for the increase of others (Mideo et al. 2008). As our study did not measure the ecological mechanisms required to definitively test the potential drivers of our spatiotemporal or land cover findings, future studies are needed to assess changes in contact between bats, contact between bats and arthropod vectors, and in bat immunity.

### 4.3 Sex and Reproductive Status, but Not Age Class, Influenced Infection Dynamics

Despite their differences in responses to spatiotemporal factors and forest loss, *Bartonella* and hemoplasma infections were similarly associated with sex and reproductive status. We found that male bats were more likely to be infected with either pathogen than female bats (Figure 3A,B). Possible drivers of this trend include the effects of testosterone on immunity and differences in behaviour between sexes. It is typically assumed that testosterone should negatively affect immune function while positively affecting characteristics important for reproduction (Folstad and Karter 1992; Muehlenbein and Bribiescas 2005). However, other studies have found the relationship between testosterone and immunity to be more complex: increased testosterone may support innate immunity over adaptive immunity and lead to behaviours that enhance transmission (Ezenwa et al. 2012; Becker, Czirják, et al. 2018). Indeed, the spread of these blood‐borne infections may be facilitated by aggressive encounters that may draw blood. While vampire bats of either sex engage in both friendly and aggressive encounters, males tend to be more aggressive and engage in fewer friendly interactions compared to females (Wilkinson 1984, 1985; Carter and Wilkinson 2013; Carter and Leffer 2015). Additionally, the *Bartonella* and hemoplasma genotypes identified here are not closely related to those found in livestock (Figures S2 and S7), suggesting that blood shared between bonded bats via regurgitation and feeding from prey is not a driver of infection.

In keeping with results from our previous work on hemoplasmas in vampire bats (Volokhov et al. 2017), reproductive bats were less likely to harbour hemoplasma infections than non‐reproductive bats (Figure 3D). This trend was also seen in our spatiotemporal models of *Bartonella* infection (Figure 3C). These findings are surprising, as reproduction is classically expected to negatively impact immunity through life history trade‐offs (Sheldon and Verhulst 1996). However, studies in bats increasingly suggest that the relationship between reproduction and immunity may be more complex (Eskew et al. 2025). In some situations, trade‐offs between immunity and reproductive functions may not occur (Ruoss et al. 2019) or reproduction may even support only certain aspects of immunity (Becker, Czirják, et al. 2018). Neither bartonellae nor hemoplasmas were influenced by age class, suggesting that bats seem to maintain susceptibility throughout their life. These results, along with instances of infection and genotype switching among recaptured bats, suggest that adaptive immunity to bartonellae and hemoplasmas may be limited at best (Figures 6 and 8). However, it is also possible that some bats switching from positive to negative infection status may be due to a reduction in bacterial loads to below levels detectable by PCR. Additionally, genotype switching may indicate loss and gain of infection or altered within‐host dynamics that allowed a less‐abundant genotype to dominate. These possible dynamics could be further elucidated through continued longitudinal study of these pathogens coupled with immune data and mathematical models, as used in the study of several bat–virus relationships (Gentles et al. 2020).

### 4.4 IBT Expectations Versus Actual Results

Past applications of IBT to infectious disease dynamics in fragmented habitats suggest that pathogens should be more common in small, isolated habitats compared to habitats that are larger and more easily accessible (Reperant 2010; Shaw et al. 2024). While our study quantified forest loss between two habitat patches as a proxy for habitat fragmentation as opposed to a direct measurement of fragmentation per se, our findings suggest that the effects of habitat loss on *Bartonella* and hemoplasma dynamics within this fragmented landscape may be more complex than what would be expected according to IBT. These findings are similar to those reported in several previous studies (Faust et al. 2018; Becker, Albery, et al. 2020). The low *R* ^2^ values from most of our models suggest that additional factors beyond those that we evaluated contribute to *Bartonella* and hemoplasma dynamics in these bats. Potentially important predictors may include edge effects of habitat fragmentation (Suzán et al. 2012), season (Field et al. 2015), ectoparasite loads and cycles (Espinal‐Palomino et al. 2025; Stuckey et al. 2017), climatic drivers (Páez et al. 2017), and the presence of alternative hosts that may dilute or enhance pathogen transmission (Ostfeld and Keesing 2000). Future studies with finer‐scale temporal sampling of vampire bats, explicit assessments of alternative transmission routes, and consideration of both edge and host community effects could provide further insight into the transmission dynamics of these bacterial pathogens under environmental change.

## 5 Conclusions

While the associations between demographic variables and infection likelihood were broadly consistent between bartonellae and hemoplasmas, our findings suggest that the effect of habitat fragmentation on bacterial infection varied across both pathogens and genotypes. Our work highlights the role that within‐pathogen variation can play in shaping population‐level transmission dynamics (Mideo et al. 2008; Withenshaw et al. 2016; Becker, Speer, et al. 2020), especially in the context of environmental change. As similar results have been found in other systems (Perrin et al. 2023), our findings add to a growing body of research that demonstrates the complexity of the ways in which habitat fragmentation can affect infectious disease dynamics in wildlife. Theories such as IBT are useful starting points for generating hypotheses about how anthropogenic change could impact the transmission of pathogens of interest, but such expectations should be explicitly tested before they are applied to any management practices.

## Author Contributions

D.J.B. and L.R.L. designed the study and interpreted results; L.R.L., K.E.D. and D.J.B. collected samples and performed lab work; D.V.V. performed lab work and data analysis; A.Y. assisted in data analysis; N.B.S. and M.B.F. coordinated fieldwork; L.R.L. analysed the data and wrote the first draft of the manuscript; all authors reviewed and approved the manuscript.

## Funding

This work was supported by the National Science Foundation (Grants DEB 1601052 and DEB 2508535), Achievement Rewards for College Scientists Foundation, Explorers Club, American Society of Mammalogists, American Museum of Natural History, National Geographic Society (Grant NGS‐55503R‐19), Research Corporation for Scientific Advancement (Grant 58‐3022‐0‐005),Edward Mallinckrodt, Jr. Foundation, and the University of Oklahoma Libraries.

## Conflicts of Interest

The authors declare no conflicts of interest.

## Supporting information


**Figure S1:** Land cover map of the region included in tree cover analyses with the extent to which the map was cropped shaded in blue. The LAR is outlined in black, and KK is circled in white. Data was acquired from the Sentinel‐2 10 m land use/land cover time series produced by Impact Observatory, Microsoft and Esri (Karra et al. 2021).
**Figure S2:** Change in tree cover in the matrix within 10 km of the central point between the LAR and KK from 2017 to 2022.
**Figure S3:** Phylogenetic tree of *Bartonella* genotypes based on *gltA* gene sequence data constructed in NGPhylogeny.fr (Lemoine et al. 2019) using maximum likelihood with smart model selection (PhyML + SMS). A threshold of 96% similarly was used to guide genotype assignments. Sequences included in this analysis are shown in blue and top BLAST hits and other relevant *Bartonella* spp. are shown in black. Nodes are coloured by bootstrap support value.
**Figure S4:** *Bartonella* genotype prevalence by year. Point estimates are shown with 95% confidence intervals (Wilson interval).
**Figure S5:** Total number of captures among recaptured vampire bats from 2015 to 2022 by the percent of bats that switched between *Bartonella* genotype (A) and the average number of unique *Bartonella* genotypes detected (B). Point estimates are shown with 95% confidence intervals (Wilson interval). Overlaid are 95% confidence bands and fitted values from the respective GLM. No significant predictors were identified for the likelihood of hemoplasma genotype switching or the number of unique hemoplasma genotypes.
**Figure S6:** Phylogenetic tree of hemoplasma genotypes based on 16S rRNA gene sequence data constructed in NGPhylogeny.fr (Lemoine et al. 2019) using maximum likelihood with smart model selection (PhyML + SMS). A threshold of 98.5% similarly was used to guide genotypes assignments. Sequences included in this analysis are shown in blue and top BLAST hits and other relevant hemoplasma spp. are shown in black. Nodes are coloured by bootstrap support value.
**Figure S7:** Prevalence of each hemoplasma genotype by year. Point estimates are shown with 95% confidence intervals (Wilson interval).

**Table S1:** Hemoplasma GenBank accession numbers for each gene sequenced.
**Table S2:** Competing suites of spatiotemporal and tree cover GLMMs for *Bartonella* and hemoplasma infection status. Within each suite, models are ranked by ΔAICc with the number of coefficients (*k*) and Akaike weights (*w*_*i*_). Dark lines indicate separations between model suites.
**Table S3:** ANOVA results of spatiotemporal GLMMs for *Bartonella* and hemoplasmas. Bolded values indicate statistical significance.
**Table S4:** Summary statistics of the top spatiotemporal GLMM for *Bartonella* and hemoplasmas. Bolded values indicate statistical significance.
**Table S5:** ANOVA results of tree cover GLMMs for *Bartonella* and hemoplasmas. Bolded values indicate statistical significance.
**Table S6:** Summary statistics of the top tree cover GLMM for *Bartonella* and hemoplasmas. Bolded values indicate statistical significance.
**Table S7:** Competing suites of spatiotemporal GLMMs for infection status per each *Bartonella* genotype. Within each suite, models are ranked by ΔAICc with the number of coefficients (*k*) and Akaike weights (*w*_*i*_). Dark lines indicate separations between model suites.
**Table S8:** ANOVA results of spatiotemporal GLMMs for *Bartonella* genogroups.
**Table S9:** Summary statistics of the top spatiotemporal GLMM for *Bartonella* genotype DR1. Bolded values indicate statistical significance.
**Table S10:** Summary statistics of the top spatiotemporal GLMM for *Bartonella* genotype DR2.
**Table S11:** Summary statistics of the top spatiotemporal GLMM for *Bartonella* genotype DR8. Bolded values indicate statistical significance.
**Table S12:** Summary statistics of the top spatiotemporal GLMM for *Bartonella* genotype DR9. Bolded values indicate statistical significance.
**Table S13:** Summary statistics of the top spatiotemporal GLMM for *Bartonella* genotype DR11. Bolded values indicate statistical significance.
**Table S14:** Competing suites of tree cover GLMMs for infection status per each *Bartonella* genotype. Within each suite, models are ranked by ΔAICc with the number of coefficients (*k*) and Akaike weights (*w*_*i*_). Dark lines indicate separations between model suites.
**Table S15:** ANOVA results of tree cover GLMMs for *Bartonella* genotypes. Bolded values indicate statistical significance.
**Table S16:** Summary statistics of the top tree cover GLMM for *Bartonella* genotype DR1. Bolded values indicate statistical significance.
**Table S17:** Summary statistics of the top tree cover GLMM for *Bartonella* genotype DR2.
**Table S18:** Summary statistics of the top tree cover GLMM for *Bartonella* genotype DR8.
**Table S19:** Summary statistics of the top tree cover GLMM for *Bartonella* genotype DR9. Bolded values indicate statistical significance.
**Table S20:** Summary statistics of the top tree cover GLMM for *Bartonella* genotype DR11.
**Table S21:** ANOVA results of GLMs for number of *Bartonella* genogroups, infection status switching and genotype switching for recaptured bats (*n* = 59). Bolded values indicate statistical significance.
**Table S22:** Summary statistics of the GLM for number of *Bartonella* genotypes in recaptured bats (*n* = 59). Bolded values indicate statistical significance.
**Table S23:** Summary statistics of the GLM for *Bartonella* infection status switching in recaptured bats (*n* = 59).
**Table S24:** Summary statistics of the GLM for *Bartonella* genotype switching in recaptured bats (*n* = 59). Bolded values indicate statistical significance.
**Table S25:** Competing suites of spatiotemporal GLMMs for infection status per each hemoplasma genotype. Within each suite, models are ranked by ΔAICc with the number of coefficients (*k*) and Akaike weights (*w*_*i*_). Dark lines indicate separations between model suites.
**Table S26:** ANOVA results of spatiotemporal GLMMs for hemoplasma genotypes. Bolded values indicate statistical significance.
**Table S27:** Summary statistics of the top spatiotemporal GLMMs for hemoplasmas genotypes. Bolded values indicate statistical significance.
**Table S28:** Competing suites of tree cover GLMMs for infection status per each hemoplasma genotype. Within each suite, models are ranked by ΔAICc with the number of coefficients (*k*) and Akaike weights (*w*_*i*_). Dark lines indicate separations between model suites.
**Table S29:** ANOVA results of tree cover GLMMs for hemoplasma genotypes.
**Table S30:** Summary statistics of the top tree cover GLMMs for hemoplasmas genotypes.
**Table S31:** ANOVA results of GLMs for number of hemoplasma genotypes, infection status switching and genotype switching for recaptured bats (*n* = 59).
**Table S32:** Summary statistics of the GLM for number of hemoplasma genotypes in recaptured bats (*n* = 59).
**Table S33:** Summary statistics of the GLM for hemoplasma infection status switching in recaptured bats (*n* = 59).
**Table S34:** Summary statistics of the GLM for hemoplasma genotype switching in recaptured bats (*n* = 59).

## Data Availability

The data that support the findings of this study are openly available in Dryad at https://doi.org/10.5061/dryad.02v6wwqg8.
