# Supplementary material for: Longitudinal Impacts of Forest Loss on Bartonella and Hemotropic Mycoplasma Dynamics in Vampire Bats Within a Fragmented Habitat
Source: Mol Ecol. 2026 Jul 11;35(13):e70466. doi: 10.1111/mec.70466 (PMC13355873; doi:10.1111/mec.70466)
Supplement: Supplementary file 1 — Figure S1: Land cover map of the region included in tree cover analyses with the extent to which the map was cropped shaded in blue. The LAR is outlined in black, and KK is circled in white. Data was acquired from the Sentinel‐2 10 m land use/land cover time series produced by Impact Observatory, Microsoft and Esri (Karra et al. 2021). Figure S2: Change in tree cover in the matrix within 10 km of the central point between the LAR and KK from 2017 to 2022. Figure S3: Phylogenetic tree of Bartonella genotypes based on gltA gene sequence data constructed in NGPhylogeny.fr (Lemoine et al. 2019) using maximum likelihood with smart model selection (PhyML + SMS). A threshold of 96% similarly was used to guide genotype assignments. Sequences included in this analysis are shown in blue and top BLAST hits and other relevant Bartonella spp. are shown in black. Nodes are coloured by bootstrap support value. Figure S4: Bartonella genotype prevalence by year. Point estimates are shown with 95% confidence intervals (Wilson interval). Figure S5: Total number of captures among recaptured vampire bats from 2015 to 2022 by the percent of bats that switched between Bartonella genotype (A) and the average number of unique Bartonella genotypes detected (B). Point estimates are shown with 95% confidence intervals (Wilson interval). Overlaid are 95% confidence bands and fitted values from the respective GLM. No significant predictors were identified for the likelihood of hemoplasma genotype switching or the number of unique hemoplasma genotypes. Figure S6: Phylogenetic tree of hemoplasma genotypes based on 16S rRNA gene sequence data constructed in NGPhylogeny.fr (Lemoine et al. 2019) using maximum likelihood with smart model selection (PhyML + SMS). A threshold of 98.5% similarly was used to guide genotypes assignments. Sequences included in this analysis are shown in blue and top BLAST hits and other relevant hemoplasma spp. are shown in black. Nodes are coloured by bootstrap support val [file MEC-35-e70466-s002.docx]

Supplemental Figures

**Longitudinal impacts of forest loss on *Bartonella* and hemotropic *Mycoplasma* dynamics in vampire bats in a fragmented habitat**

Lauren R. Lock*, Kristin E. Dyer, Dmitriy V. Volokhov, Anni Yang, M. Brock Fenton, Nancy B. Simmons, Daniel J. Becker


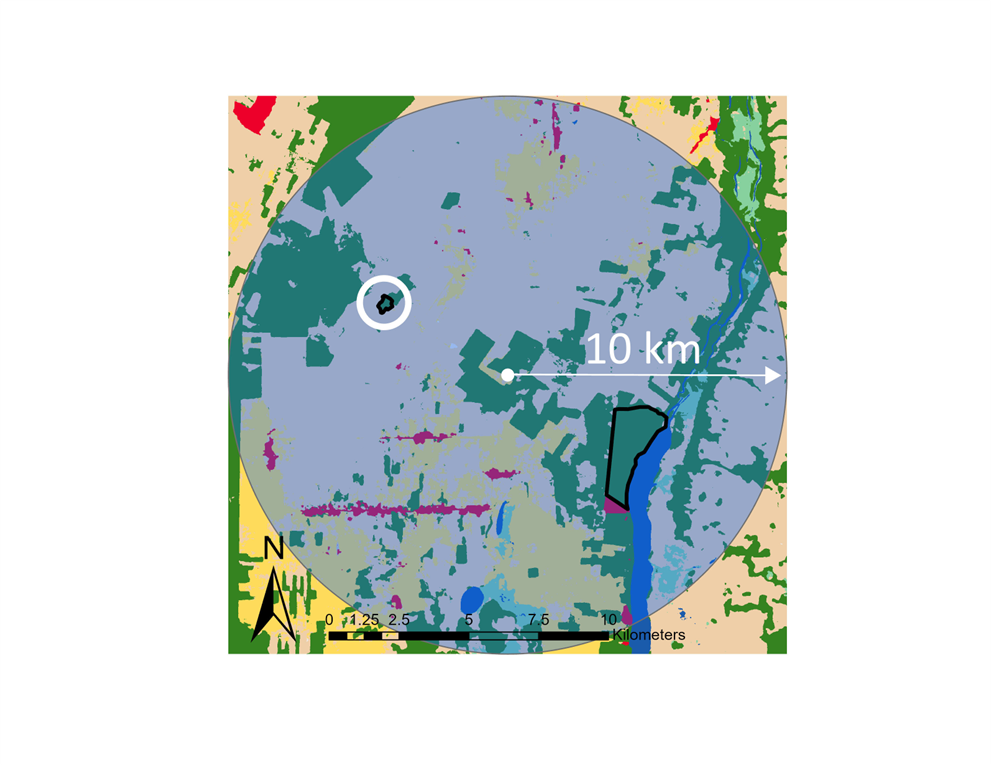


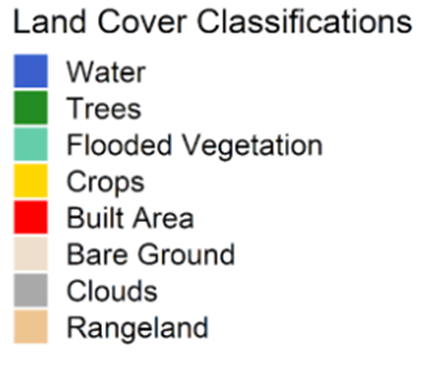


Figure S1. Land cover map of the region included in tree cover analyses with the extent to which the map was cropped shaded in blue. LAR is outlined in black, and KK is circled in white. Data was acquired from the Sentinel-2 10m Land Use/Land Cover Time Series produced by Impact Observatory, Microsoft, and Esri (Karra, Kontgis, et al. 2021).


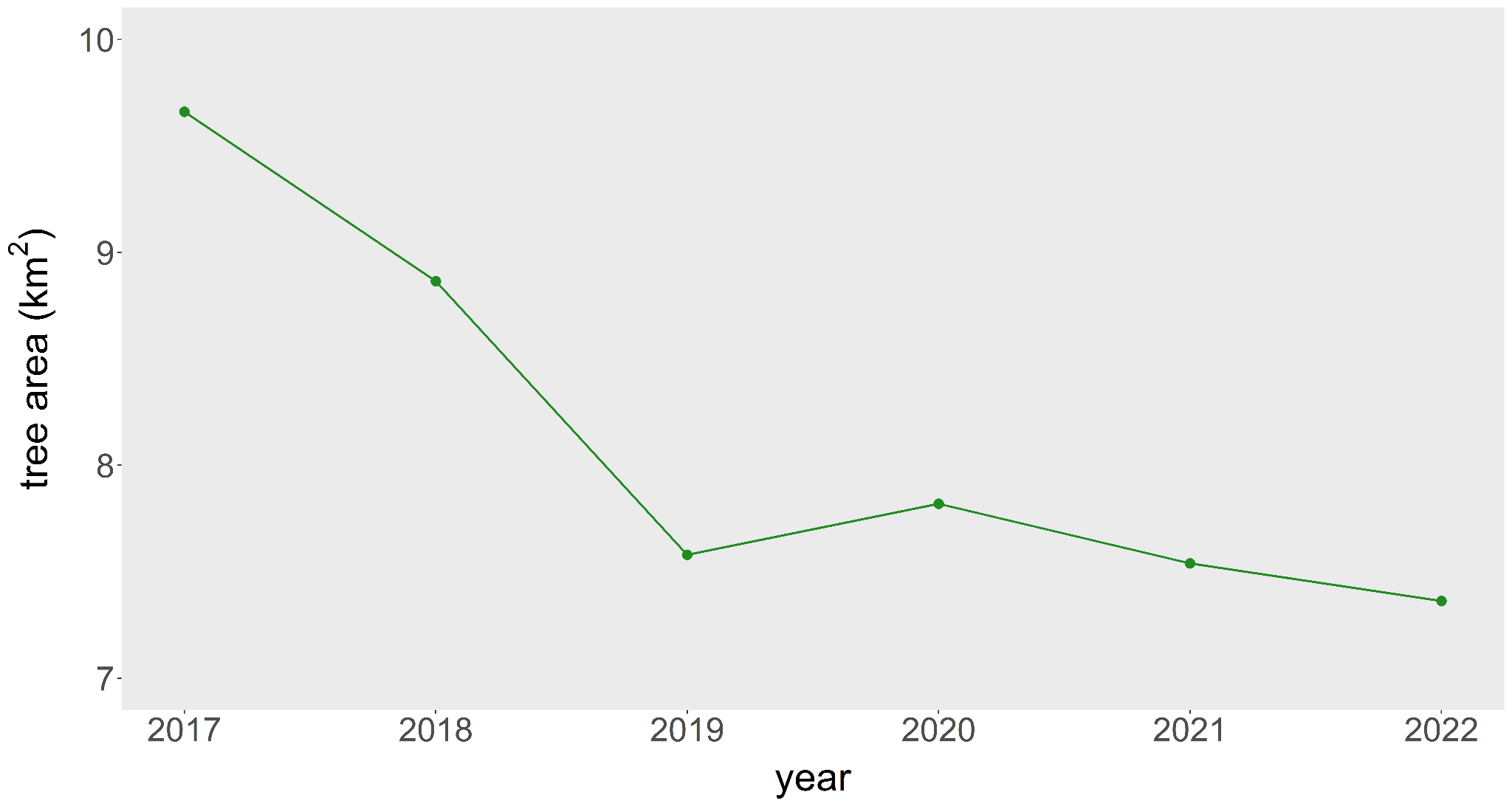


Figure S2. Change in tree cover in the matrix within 10 km of the central point between the LAR and KK from 2017-2022.


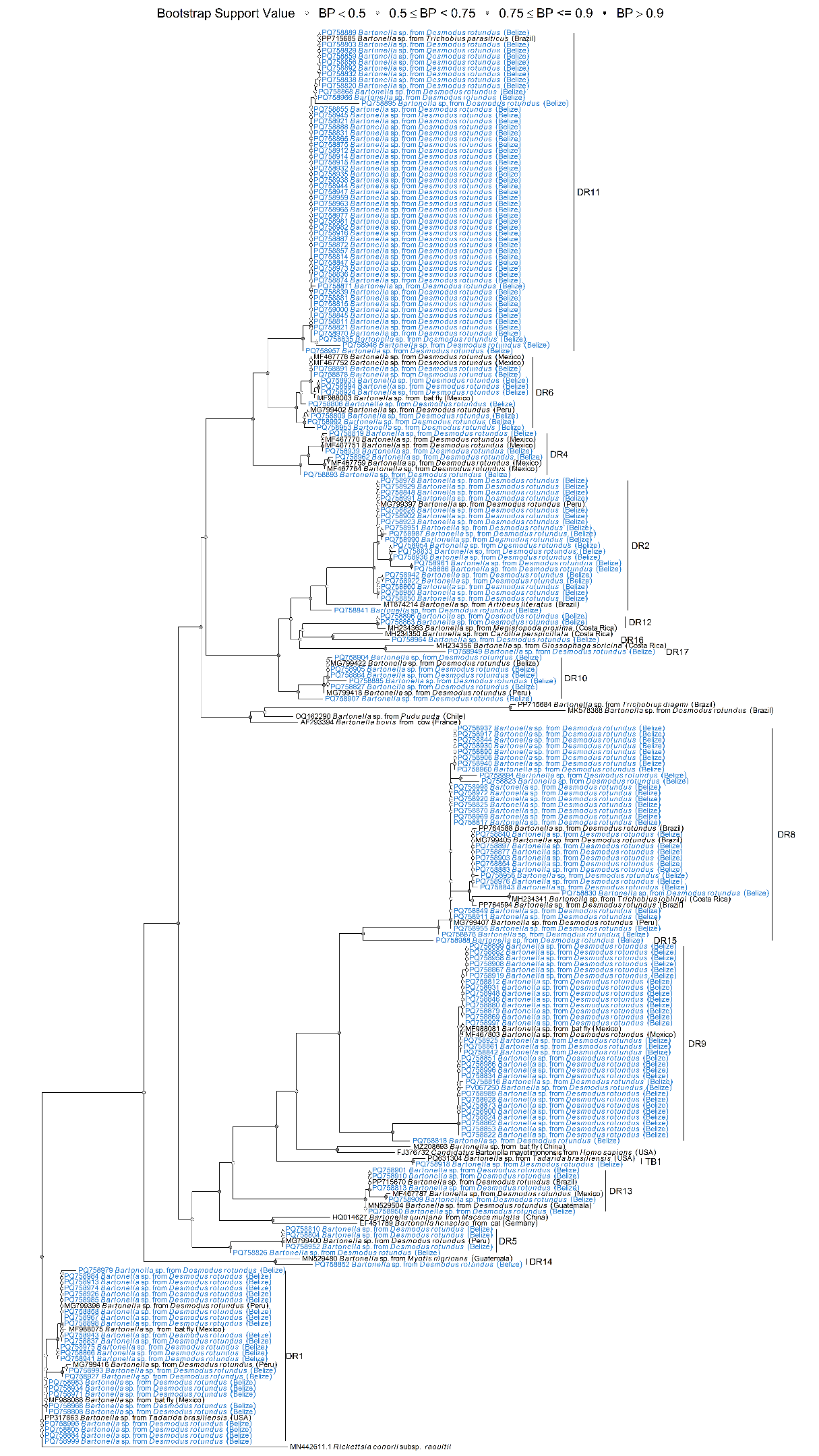


Figure S3. Phylogenetic tree of *Bartonella* genotypes based on *gltA* gene sequence data constructed in NGPhylogeny.fr (Lemoine et al., 2019) using maximum likelihood with smart model selection (PhyML + SMS). A threshold of 96% similarly was used to guide genotype assignments. Sequences included in this analysis are shown in blue and top BLAST hits and other relevant *Bartonella* spp. are shown in black. Nodes are colored by bootstrap support value.


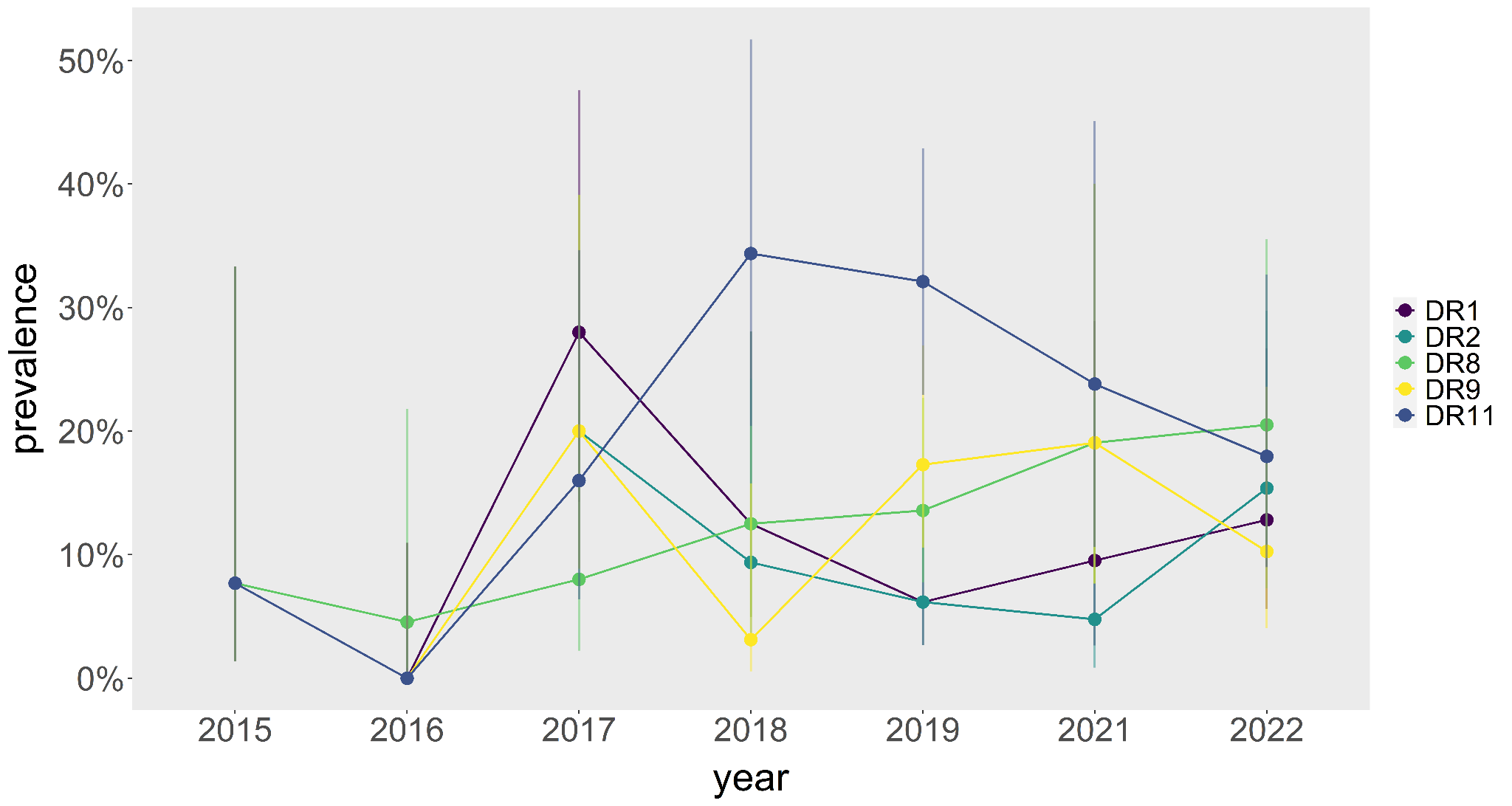


Figure S4. *Bartonella* genotype prevalence by year. Point estimates are shown with 95% confidence intervals (Wilson’s interval).


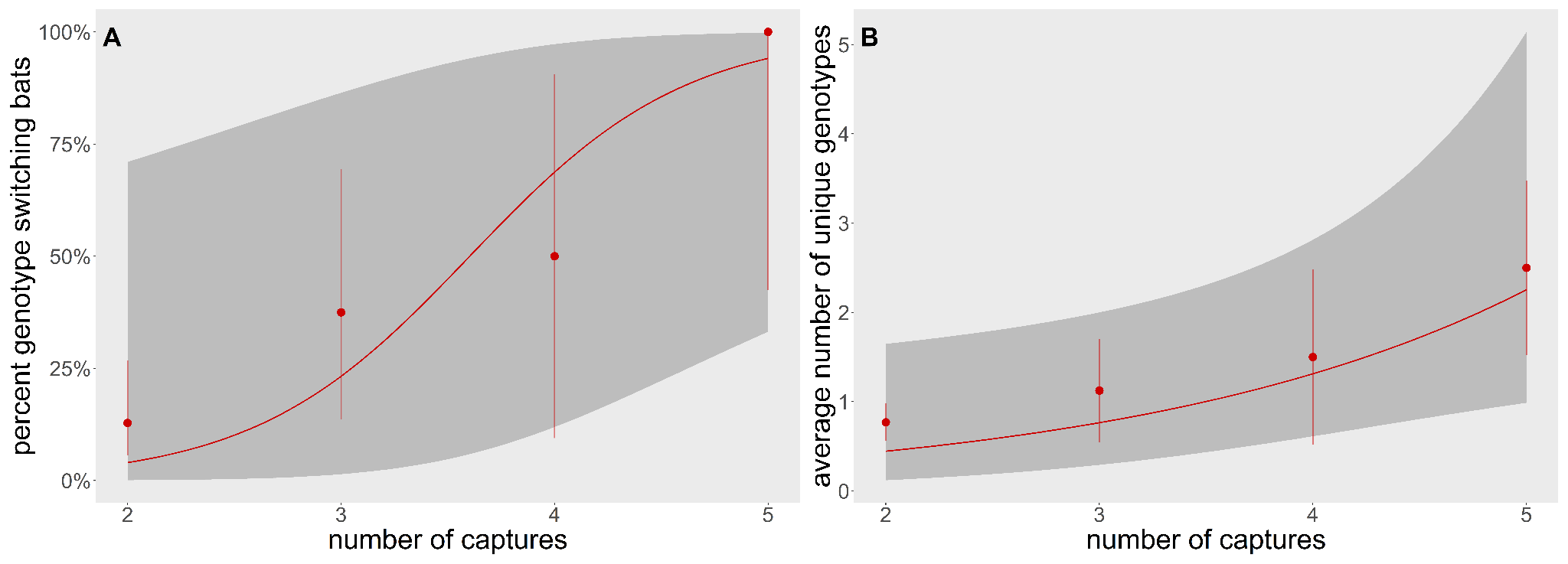


Figure S5. Total number of captures among recaptured vampire bats from 2015–2022 by the percent of bats that switched between *Bartonella* genotype (A) and the average number of unique *Bartonella* genotypes detected (B). Point estimates are shown with 95% confidence intervals (Wilson’s interval). Overlaid are 95% confidence bands and fitted values from the respective GLM. No significant predictors were identified for the likelihood of hemoplasma genotype switching or the number of unique hemoplasma genotypes.


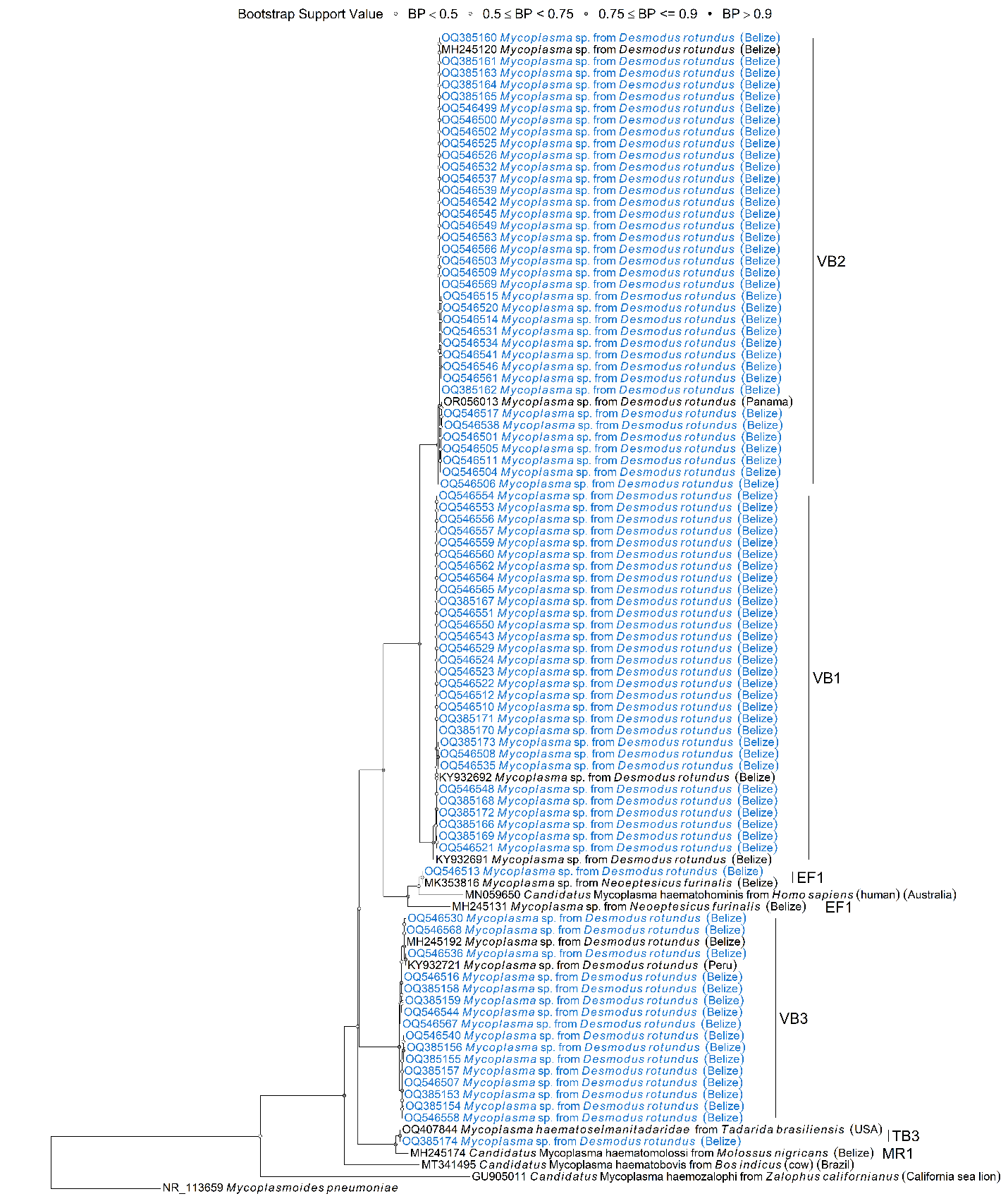


Figure S6. Phylogenetic tree of hemoplasma genotypes based on 16S rRNA gene sequence data constructed in NGPhylogeny.fr (Lemoine et al., 2019) using maximum likelihood with smart model selection (PhyML + SMS). A threshold of 98.5% similarly was used to guide genotypes assignments. Sequences included in this analysis are shown in blue and top BLAST hits and other relevant hemoplasma spp. are shown in black. Nodes are colored by bootstrap support value.


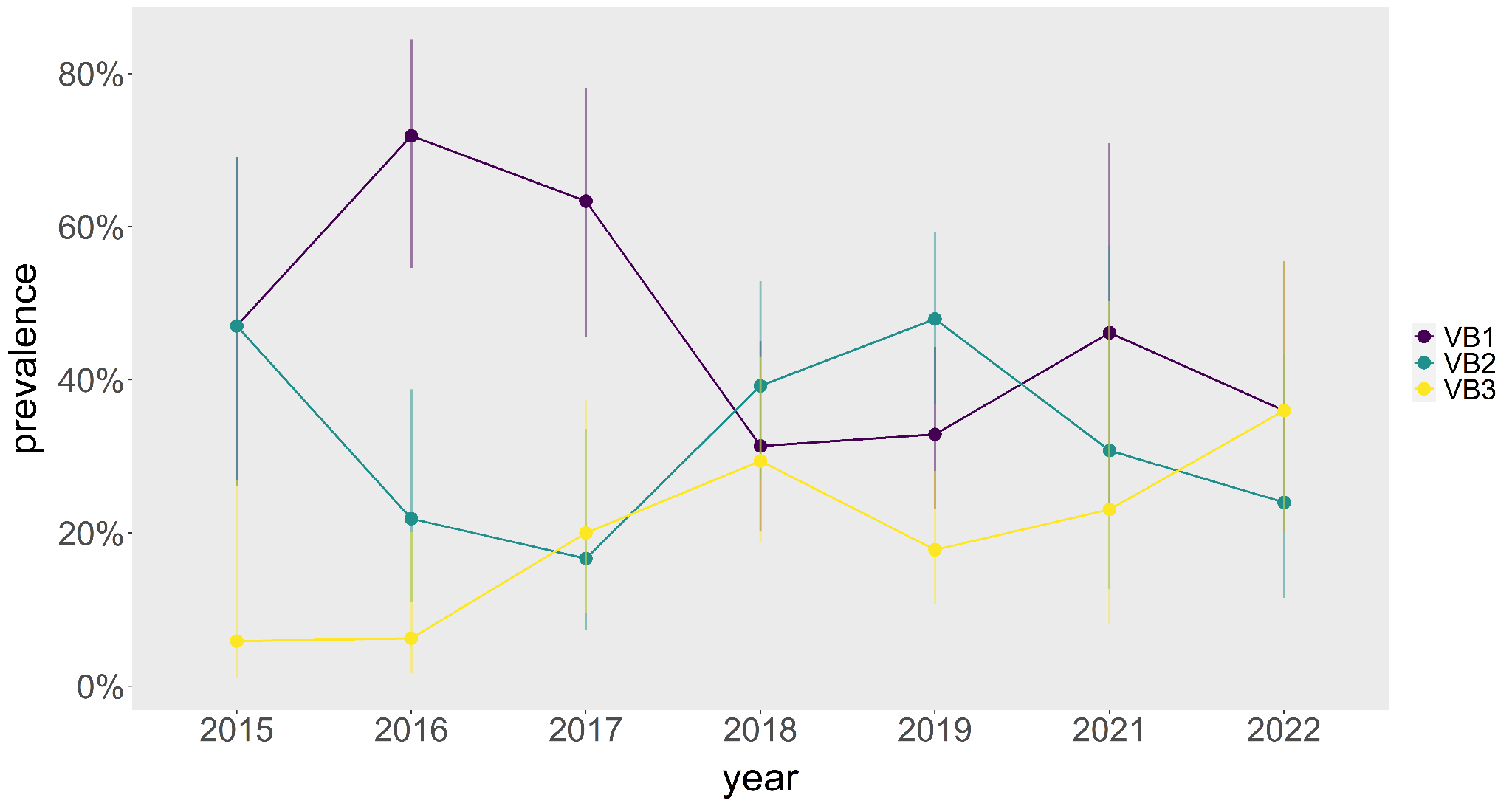


Figure S7. Prevalence of each hemoplasma genotype by year. Point estimates are shown with 95% confidence intervals (Wilson’s interval).
