# Supplementary material for: Longitudinal Impacts of Forest Loss on Bartonella and Hemotropic Mycoplasma Dynamics in Vampire Bats Within a Fragmented Habitat
Source: Mol Ecol. 2026 Jul 11;35(13):e70466. doi: 10.1111/mec.70466 (PMC13355873; doi:10.1111/mec.70466)
Supplement: Supplementary file 2 — Table S1: Hemoplasma GenBank accession numbers for each gene sequenced. Table S2: Competing suites of spatiotemporal and tree cover GLMMs for Bartonella and hemoplasma infection status. Within each suite, models are ranked by ΔAICc with the number of coefficients (k) and Akaike weights (w i ). Dark lines indicate separations between model suites. Table S3: ANOVA results of spatiotemporal GLMMs for Bartonella and hemoplasmas. Bolded values indicate statistical significance. Table S4: Summary statistics of the top spatiotemporal GLMM for Bartonella and hemoplasmas. Bolded values indicate statistical significance. Table S5: ANOVA results of tree cover GLMMs for Bartonella and hemoplasmas. Bolded values indicate statistical significance. Table S6: Summary statistics of the top tree cover GLMM for Bartonella and hemoplasmas. Bolded values indicate statistical significance. Table S7: Competing suites of spatiotemporal GLMMs for infection status per each Bartonella genotype. Within each suite, models are ranked by ΔAICc with the number of coefficients (k) and Akaike weights (w i ). Dark lines indicate separations between model suites. Table S8: ANOVA results of spatiotemporal GLMMs for Bartonella genogroups. Table S9: Summary statistics of the top spatiotemporal GLMM for Bartonella genotype DR1. Bolded values indicate statistical significance. Table S10: Summary statistics of the top spatiotemporal GLMM for Bartonella genotype DR2. Table S11: Summary statistics of the top spatiotemporal GLMM for Bartonella genotype DR8. Bolded values indicate statistical significance. Table S12: Summary statistics of the top spatiotemporal GLMM for Bartonella genotype DR9. Bolded values indicate statistical significance. Table S13: Summary statistics of the top spatiotemporal GLMM for Bartonella genotype DR11. Bolded values indicate statistical significance. Table S14: Competing suites of tree cover GLMMs for infection status per each Bartonella genotype. Within each suite, models are rank [file MEC-35-e70466-s001.docx]

Supplemental Tables

**Longitudinal impacts of forest loss on *Bartonella* and hemotropic *Mycoplasma* dynamics in vampire bats in a fragmented habitat**

Lauren R. Lock*, Kristin E. Dyer, Dmitriy V. Volokhov, Anni Yang, M. Brock Fenton, Nancy B. Simmons, Daniel J. Becker

Table S1. Hemoplasma GenBank accession numbers for each gene sequenced.

| 16S | 23S | rpoB |
| --- | --- | --- |
| KY932674.1 - KY932676.1, KY932680.1, KY932681.1, KY932685.1 - KY932700.1, KY932722.1, MH245119.1, MH245120.1, MH245123.1, MH245130.1, MH245167.1, MH245176.1- MH245181.1, MH245191.1 - MH245193.1, MK353807.1, MK353808.1, MK353815.1, MK353821.1, MK353828.1, MK353836.1, MK353839.1, MK353846.1, MK353863.1, MK353872.1, MK353880.1, MK353881.1, MK353886.1 - MK353888.1, OQ385153.1 - OQ385174.1, OQ533048.1, OQ546498.1 - OQ546569.1,  OR783317.1 - OR783319.1 | OQ456393.1,  OQ518926.1 - OQ518933.1, OQ518936.1 - OQ518942.1, OQ518945.1, OQ518946.1 | OQ554324.1, OQ554325.1, OQ554326.1, OQ554327.1 |

Table S2. Competing suites of spatiotemporal and tree cover GLMMs for *Bartonella* and hemoplasma infection status. Within each suite, models are ranked by ΔAICc with the number of coefficients (*k*) and Akaike weights (*w_i_*). Dark lines indicate separations between model suites.

| model | *k* | ΔAICc | *w_i_* |
| --- | --- | --- | --- |
| *Bartonella* infection status ~ site + year + sex + reproductive status + age class + (1\|ID) | 11 | 0 | 0.84 |
| *Bartonella* infection status ~ site + year + sex*reproductive status + sex*age class + (1\|ID) | 13 | 4.05 | 0.11 |
| *Bartonella* infection status ~ site* year + sex + reproductive status + age class + (1\|ID) | 16 | 6.39 | 0.03 |
| *Bartonella* infection status ~ site*year + sex*reproductive status + sex*age class + (1\|ID) | 18 | 8.56 | 0.01 |
| *Bartonella* infection status ~ site*tree cover + sex + reproductive status + age class+ (1\|ID) | 7 | 0 | 0.61 |
| *Bartonella* infection status ~ site + tree cover + sex + reproductive status + age class+ (1\|ID) | 6 | 2.03 | 0.22 |
| *Bartonella* infection status ~ site*tree cover + sex*reproductive status + sex*age class + (1\|ID) | 9 | 3.15 | 0.13 |
| *Bartonella* infection status ~ site + tree cover + sex*reproductive status + sex*age class + (1\|ID) | 8 | 5.06 | 0.05 |
| Hemoplasma infection status ~ site*year + sex + reproductive status + age class + (1\|ID) | 16 | 0 | 0.81 |
| Hemoplasma infection status ~ site*year + sex*reproductive status + sex*age class + (1\|ID) | 18 | 3.61 | 0.13 |
| Hemoplasma infection status ~ site + year + sex + reproductive status + age class + (1\|ID) | 11 | 5.64 | 0.05 |
| Hemoplasma infection status ~ site + year + sex*reproductive status + sex*age class + (1\|ID) | 13 | 9.41 | 0.01 |
| Hemoplasma infection status ~ site + tree cover + sex + reproductive status + age class + (1\|ID) | 6 | 0 | 0.50 |
| Hemoplasma infection status ~ site*tree cover + sex + reproductive status + age class + (1\|ID) | 7 | 0.85 | 0.33 |
| Hemoplasma infection status ~ site + tree cover + sex*reproductive status + sex*age class + (1\|ID) | 8 | 3.12 | 0.11 |
| Hemoplasma infection status ~ site*tree cover + sex*reproductive status +sex* age class + (1\|ID) | 9 | 4.03 | 0.07 |

Table S3. ANOVA results of spatiotemporal GLMMs for *Bartonella* and hemoplasmas. Bolded values indicate statistical significance.

|  | *Bartonella* | | Hemoplasmas | |
| --- | --- | --- | --- | --- |
| Fixed effect | *χ^2^* | *p* | *χ^2^* | *p* |
| site | 1.75 | 0.19 | 0.01 | 0.91 |
| year | **23.2** | **<0.001** | 3.76 | 0.71 |
| sex | **8.53** | **<0.01** | **8.58** | **<0.01** |
| reproductive status | **4.02** | **0.04** | **4.24** | **0.04** |
| age class | 0.35 | 0.55 | 1.75 | 0.19 |
| site*year | NA | NA | **13.8** | **0.02** |

Table S4. Summary statistics of the top spatiotemporal GLMM for *Bartonella* and hemoplasmas. Bolded values indicate statistical significance.

|  | *Bartonella* | | | Hemoplasmas | | |
| --- | --- | --- | --- | --- | --- | --- |
| Fixed effect | OR | *z* | *p* | OR | *z* | *p* |
| intercept | 0.54 | -1.27 | 0.20 | 0.63 | -0.79 | 0.43 |
| site (LAR-KK) | 1.44 | 1.32 | 0.19 | 5.12 | 1.63 | 0.10 |
| year (2016-2015) | 0.86 | -0.30 | 0.77 | 0.83 | -0.21 | 0.83 |
| year (2017-2015) | 2.08 | 1.34 | 0.18 | **14.87** | **2.82** | **<0.01** |
| year (2018-2015) | 0.74 | -0.62 | 0.53 | 1.53 | 0.61 | 0.54 |
| year (2019-2015) | 2.11 | 1.57 | 0.12 | 3.00 | 1.57 | 0.12 |
| year (2021-2015) | **4.56** | **2.26** | **0.02** | 0.25 | -1.48 | 0.14 |
| year (2022-2015) | **3.36** | **2.23** | **0.03** | 0.61 | -0.52 | 0.60 |
| sex (M-F) | **2.30** | **2.92** | **<0.01** | **2.38** | **2.93** | **<0.01** |
| reproductive status (Y-N) | **0.55** | **-2.01** | **0.05** | **0.54** | **-2.06** | **0.04** |
| age class (SA-A) | 1.21 | 0.59 | 0.55 | 1.57 | 1.32 | 0.19 |
| site (LAR):year (2016) | NA | NA | NA | 0.65 | -0.34 | 0.73 |
| site (LAR):year (2017) | NA | NA | NA | **0.02** | **-2.86** | **<0.01** |
| site (LAR):year (2018) | NA | NA | NA | 0.27 | -1.15 | 0.25 |
| site (LAR):year (2019) | NA | NA | NA | **0.09** | **-2.14** | **0.03** |
| site (LAR):year (2022) | NA | NA | NA | 0.41 | -0.68 | 0.50 |

Table S5. ANOVA results of tree cover GLMMs for *Bartonella* and hemoplasmas. Bolded values indicate statistical significance.

|  | *Bartonella* | | Hemoplasmas | |
| --- | --- | --- | --- | --- |
| Fixed effect | *χ^2^* | *p* | *χ^2^* | *p* |
| site | 2.01 | 0.16 | 2.71 | 0.10 |
| tree cover (increasing) | **7.22** | **<0.01** | 3.01 | 0.08 |
| sex | **10.49** | **0.001** | **7.35** | **<0.01** |
| reproductive status | 3.04 | 0.08 | **6.74** | **<0.01** |
| age class | 0.54 | 0.46 | 0.70 | 0.40 |
| site*tree cover (increasing) | **4.12** | **0.04** | NA | NA |

Table S6. Summary statistics of the top tree cover GLMM for *Bartonella* and hemoplasmas. Bolded values indicate statistical significance.

|  | *Bartonella* | | | Hemoplasmas | | |
| --- | --- | --- | --- | --- | --- | --- |
| Fixed effect | OR | *z* | *p* | OR | *z* | *p* |
| intercept | 0.65 | -0.20 | 0.84 | 0.23 | -1.12 | 0.26 |
| site (LAR-KK) | **259.25** | **2.17** | **0.03** | 0.63 | -1.65 | 0.10 |
| tree cover (increasing) | 1.02 | 0.07 | 0.95 | 1.29 | 1.74 | 0.08 |
| sex (M-F) | **2.38** | **3.24** | **<0.01** | **2.02** | **2.71** | **<0.01** |
| reproductive status (Y-N) | 0.60 | -1.74 | 0.08 | **0.47** | **-2.60** | **<0.01** |
| age class (SA-A) | 1.29 | 0.74 | 0.46 | 1.32 | 0.84 | 0.40 |
| site (LAR):tree cover (increasing) | **0.54** | **-2.03** | **0.04** | NA | NA | NA |

Table S7. Competing suites of spatiotemporal GLMMs for infection status per each *Bartonella* genotype. Within each suite, models are ranked by ΔAICc with the number of coefficients (*k*) and Akaike weights (*w_i_*). Dark lines indicate separations between model suites.

| model | *k* | ΔAICc | *w_i_* |
| --- | --- | --- | --- |
| DR1 infection status ~ site + year + sex + reproductive status + age class + (1\|ID) | 11 | 0 | 0.81 |
| DR1 infection status ~ site*year + sex + reproductive status + age class + (1\|ID) | 16 | 4.20 | 0.10 |
| DR1 infection status ~ site + year + sex* reproductive status + sex*age class + (1\|ID) | 13 | 4.61 | 0.08 |
| DR1 infection status ~ site*year + sex*reproductive status + sex*age class + (1\|ID) | 18 | 8.87 | 0.01 |
| DR2 infection status ~ site + year + sex + reproductive status + age class + (1\|ID) | 11 | 0 | 0.79 |
| DR2 infection status ~ site + year + sex* reproductive status + sex* age class + (1\|ID) | 13 | 3.96 | 0.11 |
| DR2 infection status ~ site*year + sex + reproductive status + age class + (1\|ID) | 16 | 4.40 | 0.09 |
| DR2 infection status ~ site*year + sex*reproductive status + sex*age class + (1\|ID) | 18 | 8.78 | 0.01 |
| DR8 infection status ~ site + year + sex*reproductive status + sex*age class + (1\|ID) | 13 | 0 | 0.53 |
| DR8 infection status ~ site + year + sex + reproductive status + age class + (1\|ID) | 11 | 0.65 | 0.39 |
| DR8 infection status ~ site*year + sex*reproductive status + sex*age class + (1\|ID) | 18 | 5.15 | 0.04 |
| DR8 infection status ~ site*year + sex + reproductive status + age class + (1\|ID) | 16 | 5.27 | 0.04 |
| DR9 infection status ~ site + year + sex + reproductive status + age class + (1\|ID) | 11 | 0 | 0.60 |
| DR9 infection status ~ site + year + sex* reproductive status + sex* age class + (1\|ID) | 13 | 1.12 | 0.34 |
| DR9 infection status ~ site*year + sex + reproductive status + age class + (1\|ID) | 16 | 5.42 | 0.04 |
| DR9 infection status ~ site*year + sex*reproductive status + sex*age class + (1\|ID) | 18 | 6.98 | 0.02 |
| DR11 infection status ~ site + year + sex + reproductive status + age class + (1\|ID) | 11 | 0 | 0.76 |
| DR11 infection status ~ site + year + sex*reproductive status + sex*age class + (1\|ID) | 13 | 3.10 | 0.16 |
| DR11 infection status ~ site*year + sex + reproductive status + age class + (1\|ID) | 16 | 4.71 | 0.07 |
| DR11 infection status ~ site*year + sex*reproductive status + sex*age class + (1\|ID) | 18 | 8.09 | 0.01 |

Table S8. ANOVA results of spatiotemporal GLMMs for *Bartonella* genogroups.

|  | DR1 | | DR2 | | DR8 | | DR9 | | DR11 | |
| --- | --- | --- | --- | --- | --- | --- | --- | --- | --- | --- |
| Fixed effect | *χ^2^* | *p* | *χ^2^* | *p* | *χ^2^* | *p* | *χ^2^* | *p* | *χ^2^* | *p* |
| site | 1.12 | 0.29 | 0.17 | 0.68 | 0.05 | 0.82 | 0.01 | 0.91 | 1.25 | 0.26 |
| year | 9.51 | 0.15 | 5.45 | 0.49 | 3.81 | 0.70 | 5.37 | 0.50 | 10.1 | 0.12 |
| sex | 0.30 | 0.59 | 0.75 | 0.39 | 2.97 | 0.08 | 1.26 | 0.26 | <0.01 | 0.93 |
| reproductive status | 0.07 | 0.80 | 0.03 | 0.87 | 1.44 | 0.23 | 0.17 | 0.68 | 0.61 | 0.43 |
| age class | 3.11 | 0.08 | 0.41 | 0.52 | 0.02 | 0.89 | 3.65 | 0.06 | 0.24 | 0.62 |

Table S9. Summary statistics of the top spatiotemporal GLMM for *Bartonella* genotype DR1. Bolded values indicate statistical significance.

| Fixed effect | OR | *z* | *p* |
| --- | --- | --- | --- |
| intercept | **<0.001** | **-2.25** | **0.02** |
| site (LAR-KK) | 1.92 | 1.06 | 0.29 |
| year (2016-2015) | <0.001 | -0.02 | 0.98 |
| year (2017-2015) | 4.04 | 1.18 | 0.24 |
| year (2018-2015) | 1.64 | 0.40 | 0.69 |
| year (2019-2015) | 0.48 | -0.61 | 0.54 |
| year (2021-2015) | 0.77 | -0.19 | 0.85 |
| year (2022-2015) | 1.15 | 0.11 | 0.91 |
| sex (M-F) | 0.73 | -0.54 | 0.59 |
| reproductive status (Y-N) | 1.17 | 0.26 | 0.80 |
| age class (SA-A) | 2.86 | 1.76 | 0.08 |

Table S10. Summary statistics of the top spatiotemporal GLMM for *Bartonella* genotype DR2.

| Fixed effect | OR | *z* | *p* |
| --- | --- | --- | --- |
| intercept | 0.12 | -1.84 | 0.07 |
| site (LAR-KK) | 1.28 | 0.41 | 0.68 |
| year (2016-2015) | <0.001 | < -0.01 | >0.99 |
| year (2017-2015) | 2.36 | 0.73 | 0.47 |
| year (2018-2015) | 1.02 | 0.01 | 0.99 |
| year (2019-2015) | 0.55 | -0.49 | 0.63 |
| year (2021-2015) | 0.41 | -0.58 | 0.56 |
| year (2022-2015) | 1.53 | 0.35 | 0.72 |
| sex (M-F) | 0.61 | -0.86 | 0.39 |
| reproductive status (Y-N) | 1.11 | 0.16 | 0.87 |
| age class (SA-A) | 1.51 | 0.64 | 0.52 |

Table S11. Summary statistics of the top spatiotemporal GLMM for *Bartonella* genotype DR8. Bolded values indicate statistical significance.

| Fixed effect | OR | *z* | *p* |
| --- | --- | --- | --- |
| intercept | **0.06** | **-2.37** | **0.02** |
| site (LAR-KK) | 0.88 | -0.23 | 0.82 |
| year (2016-2015) | 6.13 | 1.11 | 0.27 |
| year (2017-2015) | 1.04 | 0.03 | 0.98 |
| year (2018-2015) | 1.94 | 0.53 | 0.59 |
| year (2019-2015) | 2.18 | 0.67 | 0.50 |
| year (2021-2015) | 3.49 | 0.99 | 0.32 |
| year (2022-2015) | 3.73 | 1.11 | 0.27 |
| sex (M-F) | 2.62 | 1.72 | 0.09 |
| reproductive status (Y-N) | 0.51 | -1.20 | 0.23 |
| age class (SA-A) | 1.07 | 0.13 | 0.89 |

Table S12. Summary statistics of the top spatiotemporal GLMM for *Bartonella* genotype DR9. Bolded values indicate statistical significance.

| Fixed effect | OR | *z* | *p* |
| --- | --- | --- | --- |
| intercept | **0.09** | **-2.12** | **0.04** |
| site (LAR-KK) | 1.06 | 0.11 | 0.91 |
| year (2016-2015) | <0.001 | < -0.01 | <0.99 |
| year (2017-2015) | 2.35 | 0.72 | 0.47 |
| year (2018-2015) | 0.44 | -0.54 | 0.59 |
| year (2019-2015) | 2.82 | 0.88 | 0.38 |
| year (2021-2015) | 2.52 | 0.71 | 0.48 |
| year (2022-2015) | 1.06 | 0.05 | 0.96 |
| sex (M-F) | 1.84 | 1.12 | 0.26 |
| reproductive status (Y-N) | 0.79 | -0.41 | 0.68 |
| age class (SA-A) | 0.26 | -1.91 | 0.06 |

Table S13. Summary statistics of the top spatiotemporal GLMM for *Bartonella* genotype DR11. Bolded values indicate statistical significance.

| Fixed effect | OR | *z* | *p* |
| --- | --- | --- | --- |
| intercept | **0.10** | **-2.08** | **0.04** |
| site (LAR-KK) | 0.60 | -1.12 | 0.26 |
| year (2016-2015) | <0.001 | -0.02 | 0.98 |
| year (2017-2015) | 2.04 | 0.60 | 0.55 |
| year (2018-2015) | 7.41 | 1.73 | 0.08 |
| year (2019-2015) | 7.92 | 1.83 | 0.07 |
| year (2021-2015) | 4.74 | 1.26 | 0.21 |
| year (2022-2015) | 2.65 | 0.83 | 0.41 |
| sex (M-F) | 0.96 | -0.09 | 0.93 |
| reproductive status (Y-N) | 1.40 | 0.78 | 0.43 |
| age class (SA-A) | 0.80 | -0.49 | 0.62 |

Table S14. Competing suites of tree cover GLMMs for infection status per each *Bartonella* genotype. Within each suite, models are ranked by ΔAICc with the number of coefficients (*k*) and Akaike weights (*w_i_*). Dark lines indicate separations between model suites.

| model | *k* | ΔAICc | *w_i_* |
| --- | --- | --- | --- |
| DR1 infection status ~ site + tree cover + sex + reproductive status + age class + (1\|ID) | 6 | 0 | 0.65 |
| DR1 infection status ~ site*tree cover + sex + reproductive status + age class + (1\|ID) | 7 | 1.92 | 0.25 |
| DR1 infection status ~ site + tree cover + sex*reproductive status + sex*age class + (1\|ID) | 8 | 4.21 | 0.08 |
| DR1 infection status ~ site*tree cover + sex*reproductive status + sex*age class + (1\|ID) | 9 | 6.20 | 0.03 |
| DR2 infection status ~ site + tree cover + sex + reproductive status + age class + (1\|ID) | 6 | 0 | 0.67 |
| DR2 infection status ~ site*tree cover + sex + reproductive status + age class + (1\|ID) | 7 | 2.19 | 0.23 |
| DR2 infection status ~ site + tree cover + sex*reproductive status + sex*age class + (1\|ID) | 8 | 4.39 | 0.07 |
| DR2 infection status ~ site*tree cover + sex*reproductive status + sex*age class + (1\|ID) | 9 | 6.63 | 0.02 |
| DR8 infection status ~ site + tree cover + sex*reproductive status + sex*age class + (1\|ID) | 8 | 0 | 0.45 |
| DR8 infection status ~ site + tree cover + sex + reproductive status + age class + (1\|ID) | 6 | 0.93 | 0.28 |
| DR8 infection status ~ site*tree cover + sex*reproductive status + sex*age class + (1\|ID) | 9 | 1.95 | 0.17 |
| DR8 infection status ~ site*tree cover + sex + reproductive status + age class + (1\|ID) | 7 | 2.93 | 0.10 |
| DR9 infection status ~ site*tree cover + sex + reproductive status + age class + (1\|ID) | 7 | 0 | 0.52 |
| DR9 infection status ~ site*tree cover + sex*reproductive status + sex*age class + (1\|ID) | 9 | 0.97 | 0.32 |
| DR9 infection status ~ site + tree cover + sex + reproductive status + age class + (1\|ID) | 6 | 3.41 | 0.10 |
| DR9 infection status ~ site + tree cover + sex*reproductive status + sex*age class + (1\|ID) | 8 | 4.35 | 0.06 |
| DR11 infection status ~ site*tree cover + sex + reproductive status + age class + (1\|ID) | 7 | 0 | 0.39 |
| DR11 infection status ~ site + tree cover + sex + reproductive status + age class + (1\|ID) | 6 | 0.18 | 0.35 |
| DR11 infection status ~ site*tree cover + sex*reproductive status + sex*age class + (1\|ID) | 9 | 2.09 | 0.14 |
| DR11 infection status ~ site + tree cover + sex*reproductive status + sex*age class + (1\|ID) | 8 | 2.27 | 0.12 |

Table S15. ANOVA results of tree cover GLMMs for *Bartonella* genotypes. Bolded values indicate statistical significance.

|  | DR1 | | DR2 | | DR8 | | DR9 | | DR11 | |
| --- | --- | --- | --- | --- | --- | --- | --- | --- | --- | --- |
| Fixed effect | *χ^2^* | *p* | *χ^2^* | *p* | *χ^2^* | *p* | *χ^2^* | *p* | *χ^2^* | *p* |
| site | 0.59 | 0.44 | <0.001 | 0.98 | 0.02 | 0.88 | 0.09 | 0.76 | 2.40 | 0.12 |
| tree cover (increasing) | **6.69** | **0.01** | 1.51 | 0.22 | 1.44 | 0.23 | 0.13 | 0.72 | 0.94 | 0.33 |
| sex | 0.22 | 0.64 | 0.54 | 0.46 | 2.99 | 0.08 | 1.30 | 0.25 | 0.52 | 0.47 |
| reproductive status | 0.26 | 0.61 | 0.19 | 0.66 | 1.39 | 0.24 | 0.49 | 0.48 | 0.16 | 0.69 |
| age class | 2.07 | 0.15 | 0.05 | 0.82 | 0.02 | 0.90 | 3.54 | 0.06 | 0.01 | 0.92 |
| site*tree cover (increasing) | NA | NA | NA | NA | NA | NA | **4.24** | **0.04** | NA | NA |

Table S16. Summary statistics of the top tree cover GLMM for *Bartonella* genotype DR1. Bolded values indicate statistical significance.

| Fixed effect | OR | *z* | *p* |
| --- | --- | --- | --- |
| intercept | **<0.001** | **-3.17** | **<0.01** |
| site (LAR-KK) | 1.61 | 0.77 | 0.44 |
| tree cover (increasing) | **2.05** | **2.59** | **0.01** |
| sex (M-F) | 0.77 | -0.47 | 0.64 |
| reproductive status (Y-N) | 1.35 | 0.51 | 0.61 |
| age class (SA-A) | 2.37 | 1.44 | 0.15 |

Table S17. Summary statistics of the top tree cover GLMM for *Bartonella* genotype DR2.

| Fixed effect | OR | *z* | *p* |
| --- | --- | --- | --- |
| intercept | <0.01 | -1.83 | 0.07 |
| site (LAR-KK) | 0.99 | -0.02 | 0.98 |
| tree cover (increasing) | 1.43 | 1.23 | 0.22 |
| sex (M-F) | 0.66 | -0.73 | 0.46 |
| reproductive status (Y-N) | 1.31 | 0.44 | 0.66 |
| age class (SA-A) | 1.16 | 0.23 | 0.82 |

Table S18. Summary statistics of the top tree cover GLMM for *Bartonella* genotype DR8.

| Fixed effect | OR | *z* | *p* |
| --- | --- | --- | --- |
| intercept | 2.72 | 0.35 | 0.73 |
| site (LAR-KK) | 1.10 | 0.15 | 0.88 |
| tree cover (increasing) | 0.68 | -1.20 | 0.23 |
| sex (M-F) | 2.63 | 1.73 | 0.08 |
| reproductive status (Y-N) | 0.52 | -1.18 | 0.24 |
| age class (SA-A) | 1.07 | 0.13 | 0.90 |

Table S19. Summary statistics of the top tree cover GLMM for *Bartonella* genotype DR9. Bolded values indicate statistical significance.

| Fixed effect | OR | *z* | *p* |
| --- | --- | --- | --- |
| intercept | <0.01 | -1.78 | 0.08 |
| site (LAR-KK) | **1.06e+6** | **2.01** | **0.04** |
| tree cover (increasing) | 2.79 | 1.54 | 0.12 |
| sex (M-F) | 1.84 | 1.14 | 0.25 |
| reproductive status (Y-N) | 0.67 | -0.70 | 0.48 |
| age class (SA-A) | 0.26 | -1.89 | 0.06 |
| Site (LAR)*tree cover increasing) | **0.20** | **-2.06** | **0.04** |

*very large OR due to positivity and sample size imbalance between sites (n_LAR_ = 23/145, n_KK_ = 5/37)

Table S20. Summary statistics of the top tree cover GLMM for *Bartonella* genotype DR11.

| Fixed effect | OR | *z* | *p* |
| --- | --- | --- | --- |
| intercept | 4.22 | 0.71 | 0.48 |
| site (LAR-KK) | 0.51 | -1.55 | 0.12 |
| tree cover (increasing) | 0.80 | -0.97 | 0.33 |
| sex (M-F) | 0.75 | -0.72 | 0.47 |
| reproductive status (Y-N) | 1.19 | 0.40 | 0.69 |
| age class (SA-A) | 0.95 | -0.11 | 0.92 |

Table S21. ANOVA results of GLMs for number of *Bartonella* genogroups, infection status switching, and genotype switching for recaptured bats (n = 59). Bolded values indicate statistical significance.

|  | Number of genotypes (Poisson GLM) | | Infection status switching (binomial GLM) | | Genotype switching (binomial GLM) | |
| --- | --- | --- | --- | --- | --- | --- |
| Fixed effect | *χ^2^* | *p* | *χ^2^* | *p* | *χ^2^* | *p* |
| number of captures | **5.36** | **0.02** | 0.19 | 0.66 | **8.13** | **<0.01** |
| sex | 1.06 | 0.30 | 1.0 | 0.32 | 1.93 | 0.17 |
| minimum age (years) | 1.02 | 0.31 | 0.00 | 1.0 | 0.81 | 0.37 |
| site | 0.27 | 0.60 | 0.13 | 0.71 | 0.69 | 0.40 |
| age class switching | 0.69 | 0.41 | 1.14 | 0.29 | 0.33 | 0.57 |

Table S22. Summary statistics of the GLM for number of *Bartonella* genotypes in recaptured bats (n = 59). Bolded values indicate statistical significance.

| Fixed effect | OR | *z* | *p* |
| --- | --- | --- | --- |
| intercept | 0.36 | -1.50 | 0.13 |
| number of captures | **1.72** | **2.25** | **0.02** |
| sex (M-F) | 1.37 | 1.02 | 0.31 |
| minimum age (years) | 0.85 | -0.97 | 0.33 |
| site (LAR-KK) | 0.72 | -0.54 | 0.59 |
| age class switching (switching-nonswitching) | 1.32 | 0.84 | 0.40 |

Table S23. Summary statistics of the GLM for *Bartonella* infection status switching in recaptured bats (n = 59).

| Fixed effect | OR | *z* | *p* |
| --- | --- | --- | --- |
| intercept | 0.39 | -0.62 | 0.53 |
| number of captures | 1.24 | 0.43 | 0.67 |
| sex (M-F) | 0.54 | -0.99 | 0.32 |
| minimum age (years) | 1.00 | 0.00 | 1.0 |
| site (LAR-KK) | 1.60 | 0.36 | 0.72 |
| age class switching (switching-nonswitching) | 2.13 | 1.05 | 0.29 |

Table S24. Summary statistics of the GLM for *Bartonella* genotype switching in recaptured bats (n = 59). Bolded values indicate statistical significance.

| Fixed effect | OR | *z* | *p* |
| --- | --- | --- | --- |
| intercept | <0.001 | -0.01 | 0.99 |
| number of captures | **7.26** | **2.28** | **0.02** |
| sex (M-F) | 3.43 | 1.32 | 0.19 |
| minimum age (years) | 0.64 | -0.84 | 0.40 |
| site (LAR-KK) | 5.34e+6* | 0.01 | 0.99 |
| age class switching (switching-nonswitching) | 1.69 | 0.58 | 0.56 |

*very large OR due to sample size imbalance between sites (n_LAR_ = 48, n_KK_ = 3)

Table S25. Competing suites of spatiotemporal GLMMs for infection status per each hemoplasma genotype. Within each suite, models are ranked by ΔAICc with the number of coefficients (*k*) and Akaike weights (*w_i_*). Dark lines indicate separations between model suites.

| model | *k* | ΔAICc | *w_i_* |
| --- | --- | --- | --- |
| VB1 infection status ~ site + year + sex + reproductive status + age class + (1\|ID) | 11 | 0 | 0.79 |
| VB1 infection status ~ site*year + sex + reproductive status + sex + age class + (1\|ID) | 16 | 4.05 | 0.10 |
| VB1 infection status ~ site + year + sex*reproductive status + sex*age class + (1\|ID) | 13 | 4.29 | 0.09 |
| VB1 infection status ~ site*year+ sex* reproductive status + sex*age class + (1\|ID) | 18 | 8.66 | 0.01 |
| VB2 infection status ~ site + year + sex + reproductive status + age class + (1\|ID) | 11 | 0 | 0.44 |
| VB2 infection status ~ site + year + sex*reproductive status + sex*age class + (1\|ID) | 13 | 0.42 | 0.36 |
| VB2 infection status ~ site*year + sex + reproductive status + age class + (1\|ID) | 16 | 2.35 | 0.14 |
| VB2 infection status ~ site*year + sex*reproductive status + sex*age class + (1\|ID) | 18 | 3.85 | 0.06 |
| VB3 infection status ~ site + year + sex + reproductive status + age class + (1\|ID) | 11 | 0 | 0.63 |
| VB3 infection status ~ site + year + sex*reproductive status + sex*age class + (1\|ID) | 13 | 1.26 | 0.34 |
| VB3 infection status ~ site*year + sex + reproductive status + age class + (1\|ID) | 16 | 6.42 | 0.03 |
| VB3 infection status ~ site*year + sex*reproductive status + sex*age class + (1\|ID) | 18 | 8.25 | 0.01 |

Table S26. ANOVA results of spatiotemporal GLMMs for hemoplasma genotypes. Bolded values indicate statistical significance.

|  | VB1 | | VB2 | | VB3 | |
| --- | --- | --- | --- | --- | --- | --- |
| Fixed effect | *χ^2^* | *p* | *χ^2^* | *p* | *χ^2^* | *p* |
| site | <0.01 | 0.93 | 0.78 | 0.38 | 0.56 | 0.45 |
| year | **23.1** | **<0.001** | **18.8** | **<0.01** | 11.5 | 0.07 |
| sex | 0.90 | 0.34 | 2.50 | 0.11 | <0.001 | 0.99 |
| reproductive status | 0.43 | 0.51 | 3.62 | 0.06 | 3.14 | 0.08 |
| age class | **3.86** | **0.05** | 1.25 | 0.26 | 1.50 | 0.22 |

Table S27. Summary statistics of the top spatiotemporal GLMMs for hemoplasmas genotypes. Bolded values indicate statistical significance.

|  | VB1 | | | VB2 | | | VB3 | | |
| --- | --- | --- | --- | --- | --- | --- | --- | --- | --- |
| Fixed effect | OR | *z* | *p* | OR | *z* | *p* | OR | *z* | *p* |
| intercept | 1.09 | 0.13 | 0.90 | 0.32 | -1.73 | 0.08 | 0.15 | -1.71 | 0.09 |
| site (LAR-KK) | 0.97 | -0.09 | 0.93 | 1.37 | 0.88 | 0.38 | 0.73 | 0.75 | 0.45 |
| year (2016-2015) | 3.04 | 1.70 | 0.09 | 0.29 | -1.83 | 0.07 | 1.05 | 0.04 | 0.97 |
| year (2017-2015) | 2.30 | 1.30 | 0.19 | **0.20** | **-2.27** | **0.02** | 3.56 | 1.12 | 0.26 |
| year (2018-2015) | 0.45 | -1.29 | 0.20 | 0.87 | -0.24 | 0.81 | 6.18 | 1.67 | 0.10 |
| year (2019-2015) | 0.49 | -1.24 | 0.21 | 1.25 | 0.39 | 0.69 | 3.23 | 1.08 | 0.28 |
| year (2021-2015) | 0.98 | -0.03 | 0.98 | 0.47 | -0.93 | 0.35 | 4.91 | 1.27 | 0.20 |
| year (2022-2015) | 0.65 | -0.64 | 0.53 | 0.30 | -1.68 | 0.09 | **9.92** | **2.03** | **0.04** |
| sex (M-F) | 0.71 | -0.95 | 0.34 | 1.79 | 1.58 | 0.11 | 1.00 | -0.01 | 0.99 |
| reproductive status (Y-N) | 0.78 | -0.65 | 0.51 | 2.09 | 1.90 | 0.06 | 0.46 | -1.77 | 0.08 |
| age class (SA-A) | **2.17** | **1.97** | **0.05** | 0.63 | -1.12 | 0.26 | 0.54 | -1.22 | 0.22 |

Table S28. Competing suites of tree cover GLMMs for infection status per each hemoplasma genotype. Within each suite, models are ranked by ΔAICc with the number of coefficients (*k*) and Akaike weights (*w_i_*). Dark lines indicate separations between model suites.

| model | *k* | ΔAICc | *w_i_* |
| --- | --- | --- | --- |
| VB1 infection status ~ site*tree cover + sex + reproductive status + age class + (1\|ID) | 7 | 0 | 0.56 |
| VB1 infection status ~ site + tree cover + sex + reproductive status + age class + (1\|ID) | 6 | 0.99 | 0.34 |
| VB1 infection status ~ site*tree cover + sex*reproductive status + sex*age class + (1\|ID) | 9 | 4.44 | 0.06 |
| VB1 infection status ~ site + tree cover + sex*reproductive status + sex*age class + (1\|ID) | 8 | 5.37 | 0.04 |
| VB2 infection status ~ site*tree cover + sex + reproductive status + age class + (1\|ID) | 7 | 0 | 0.65 |
| VB2 infection status ~ site*tree cover + sex*reproductive status +sex*age class + (1\|ID) | 9 | 1.62 | 0.29 |
| VB2 infection status ~ site + tree cover + sex + reproductive status + age class + (1\|ID) | 6 | 5.50 | 0.04 |
| VB2 infection status ~ site + tree cover + sex*reproductive status + sex*age class + (1\|ID) | 8 | 6.81 | 0.02 |
| VB3 infection status ~ site + tree cover + sex + reproductive status + age class + (1\|ID) | 6 | 0 | 0.37 |
| VB3 infection status ~ site*tree cover + sex + reproductive status + age class + (1\|ID) | 7 | 0.41 | 0.30 |
| VB3 infection status ~ site + tree cover + sex*rep + sex*age class + (1\|ID) | 8 | 1.42 | 0.18 |
| VB3 infection status ~ site*tree cover + sex*reproductive status + sex*age class + (1\|ID) | 9 | 1.92 | 0.14 |

Table S29. ANOVA results of tree cover GLMMs for hemoplasma genotypes.

|  | VB1 | | VB2 | | VB3 | |
| --- | --- | --- | --- | --- | --- | --- |
| Fixed effect | χ^2^ | *p* | χ^2^ | *p* | χ^2^ | *p* |
| site | 0.23 | 0.63 | 0.01 | 0.91 | 0.08 | 0.78 |
| tree cover | **4.59** | **0.03** | 2.55 | 0.11 | 0.00 | 1.0 |
| sex | 0.06 | 0.81 | 0.62 | 0.43 | <0.01 | 0.95 |
| reproductive status | 0.03 | 0.87 | 1.50 | 0.22 | 2.46 | 0.12 |
| age class | 2.83 | 0.09 | 0.67 | 0.41 | 0.98 | 0.32 |
| site:tree cover | NA | NA | **7.17** | **<0.01** | NA | NA |

Table S30. Summary statistics of the top tree cover GLMMs for hemoplasmas genotypes.

|  | VB1 | | | VB2 | | | VB3 | | |
| --- | --- | --- | --- | --- | --- | --- | --- | --- | --- |
| Fixed effect | OR | *z* | *p* | OR | *z* | *p* | OR | *z* | *p* |
| intercept | **0.02** | **-2.36** | **0.02** | **406** | **2.76** | **<0.01** | 0.58 | -0.28 | 0.78 |
| site (LAR-KK) | 1.20 | 0.48 | 0.63 | **<0.001** | **-2.67** | **<0.01** | 0.89 | -0.28 | 0.78 |
| tree cover (increasing) | **1.51** | **2.14** | **0.03** | **0.33** | **-3.11** | **<0.01** | 1.00 | 0.00 | 1.00 |
| sex (M-F) | 0.91 | -0.24 | 0.81 | 1.34 | 0.79 | 0.43 | 0.97 | -0.07 | 0.95 |
| reproductive status (Y-N) | 0.94 | -0.16 | 0.87 | 1.64 | 1.22 | 0.22 | 0.50 | -1.57 | 0.18 |
| age class (SA-A) | 2.06 | 1.68 | 0.09 | 0.69 | -0.82 | 0.41 | 0.61 | 0.99 | 0.32 |
| site (LAR):tree cover (increasing) | NA | NA | NA | **3.18** | **2.68** | **<0.01** | NA | NA | NA |

Table S31. ANOVA results of GLMs for number of hemoplasma genotypes, infection status switching, and genotype switching for recaptured bats (n = 59).

|  | Number of genotypes  (Poisson GLM) | | Infection status switching (binomial GLM) | | Genotype switching (binomial GLM) | |
| --- | --- | --- | --- | --- | --- | --- |
| Fixed effect | *χ^2^* | *p* | *χ^2^* | *p* | *χ^2^* | *p* |
| number of captures | 0.01 | 0.90 | 1.22 | 0.27 | 1.07 | 0.30 |
| sex | 0.03 | 0.86 | 0.09 | 0.77 | 0.08 | 0.78 |
| minimum age (years) | 0.04 | 0.85 | 0.28 | 0.60 | 0.32 | 0.57 |
| site | 0.01 | 0.91 | 0.31 | 0.58 | 1.97 | 0.16 |
| age class switching | 0.69 | 0.41 | 0.65 | 0.42 | <0.01 | 0.94 |

Table S32. Summary statistics of the GLM for number of hemoplasma genotypes in recaptured bats (n = 59).

| Fixed effect | OR | *z* | *p* |
| --- | --- | --- | --- |
| intercept | 0.97 | -0.04 | 0.97 |
| number of captures | 1.03 | 0.12 | 0.90 |
| sex (M-F) | 1.05 | 0.18 | 0.86 |
| minimum age (years) | 0.98 | -0.19 | 0.85 |
| site (LAR-KK) | 0.94 | -0.12 | 0.91 |
| age class switching (switching-nonswitching) | 1.32 | 0.84 | 0.40 |

Table S33. Summary statistics of the GLM for hemoplasma infection status switching in recaptured bats (n = 59).

| Fixed effect | OR | *z* | *p* |
| --- | --- | --- | --- |
| intercept | 0.30 | -0.91 | 0.37 |
| number of captures | 1.69 | 1.04 | 0.30 |
| sex (M-F) | 0.83 | -0.30 | 0.77 |
| minimum age (years) | 1.15 | 0.53 | 0.59 |
| site (LAR-KK) | 0.54 | -0.56 | 0.57 |
| age class switching (switching-nonswitching) | 1.82 | 0.81 | 0.42 |

Table S34. Summary statistics of the GLM for hemoplasma genotype switching in recaptured bats (n = 59).

| Fixed effect | OR | *z* | *p* |
| --- | --- | --- | --- |
| intercept | <0.001 | -0.01 | 0.99 |
| number of captures | 1.65 | 1.01 | 0.31 |
| sex (M-F) | 0.82 | -0.28 | 0.78 |
| minimum age (years) | 0.83 | -0.54 | 0.59 |
| site (LAR-KK) | 1.29e+7* | 0.01 | 0.99 |
| age class switching (switching-nonswitching) | 1.07 | 0.08 | 0.94 |

*very large OR due to sample size imbalance between sites (n_LAR_ = 48, n_KK_ = 4)
